# Wide-Ranging Effects on the Brain Proteome in a Transgenic Mouse Model of Alzheimer’s Disease Following Treatment with a Brain-Targeting Somatostatin Peptide

**DOI:** 10.1021/acschemneuro.1c00303

**Published:** 2021-06-25

**Authors:** Fadi Rofo, Friederike A. Sandbaumhüter, Aikaterini Chourlia, Nicole G. Metzendorf, Jamie I. Morrison, Stina Syvänen, Per E. Andrén, Erik T. Jansson, Greta Hultqvist

**Affiliations:** † Protein Drug Design, Faculty of Pharmacy, Biomedical Centre 591, Uppsala University, 75124 Uppsala, Sweden; ‡ Medical Mass Spectrometry, Department of Pharmaceutical Biosciences, Biomedical Centre 591, Uppsala University, 75124 Uppsala, Sweden; § Department of Public Health and Caring Sciences, Rudbeck Laboratory, Uppsala University, 75185 Uppsala, Sweden; ∥ Science for Life Laboratory, Spatial Mass Spectrometry, Biomedical Centre 591, Uppsala University, 75124 Uppsala, Sweden

**Keywords:** Alzheimer’s disease, amyloid-β, proteomics, somatostatin, SST-scFv8D3, LC−MS

## Abstract

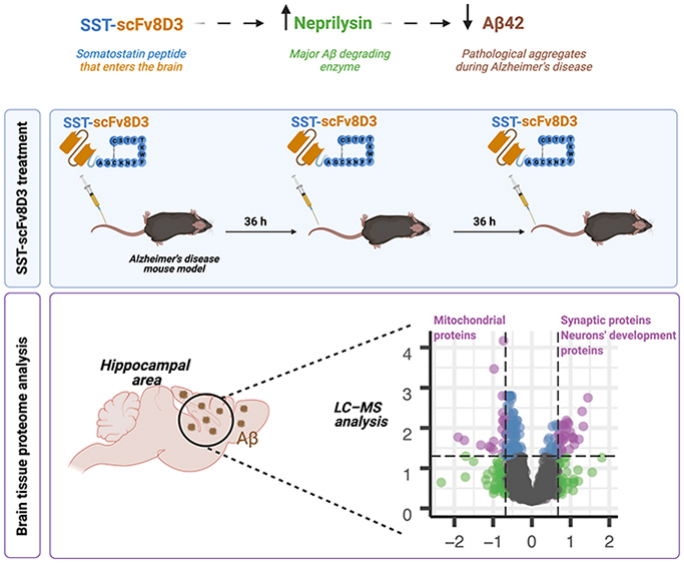

Alzheimer’s disease is the most common neurodegenerative disorder characterized by the pathological aggregation of amyloid-β (Aβ) peptide. A potential therapeutic intervention in Alzheimer’s disease is to enhance Aβ degradation by increasing the activity of Aβ-degrading enzymes, including neprilysin. The somatostatin (SST) peptide has been identified as an activator of neprilysin. Recently, we demonstrated the ability of a brain-penetrating SST peptide (SST-scFv8D3) to increase neprilysin activity and membrane-bound Aβ42 degradation in the hippocampus of mice overexpressing the Aβ-precursor protein with the Swedish mutation (APPswe). Using LC–MS, we further evaluated the anti-Alzheimer’s disease effects of SST-scFv8D3. Following a triple intravenous injection of SST-scFv8D3, the LC–MS analysis of the brain proteome revealed that the majority of downregulated proteins consisted of mitochondrial proteins regulating fatty acid oxidation, which are otherwise upregulated in APPswe mice compared to wild-type mice. Moreover, treatment with SST-scFv8D3 significantly increased hippocampal levels of synaptic proteins regulating cell membrane trafficking and neuronal development. Finally, hippocampal concentrations of growth-regulated α (KC/GRO) chemokine and degradation of neuropeptide-Y were elevated after SST-scFv8D3 treatment. In summary, our results demonstrate a multifaceted effect profile in regulating mitochondrial function and neurogenesis following treatment with SST-scFv8D3, further suggesting the development of Alzheimer’s disease therapies based on SST peptides.

## Introduction

Alzheimer’s disease is a progressive and multifactorial neurodegenerative disorder. It is also the most common cause of dementia. In the brain of Alzheimer’s patients, extracellular amyloid aggregates and intracellular neurofibrillary tangles are formed, leading to synaptic toxicity and neuronal death.^1^ The pathological amyloid aggregation is formed by a 36–43 amino acid long peptide called amyloid-β (Aβ). The latter is generated by enzymatic digestion of the transmembrane protein, amyloid precursor protein (APP). Familial mutations in APP, such as the Swedish mutation (APPswe) (KM670/671NL), increase Aβ production and lead to early onset of Alzheimer’s disease.^2^ Accumulation of Aβ can also be attributed to the decline in the activity and/or levels of the Aβ-degrading enzymes, including neprilysin.^3^

Neprilysin is a membrane-bound zinc–metallopeptidase with a large extracellular domain containing the catalytic site. The enzyme has displayed the ability to degrade Aβ but not its precursor APP.^4^ The protein levels of neprilysin are downregulated during aging and Alzheimer’s disease, both in transgenic mouse models of Alzheimer’s disease and in post-mortem human brains.^5,6^ Accordingly, molecules that enhance the activity or levels of neprilysin are considered as potential therapeutic options for the treatment of Alzheimer’s disease.

A major activator of neprilysin is somatostatin (SST), a cyclic neuropeptide involved in processes of neuromodulation and cell proliferation through its interaction with SST receptors (SSTRs).^7^ Importantly, SST has been identified as a major activator of neprilysin. Treatment of primary neuronal cultures with this peptide has been associated with enhanced neprilysin activity and decreased Aβ42 concentration.^8^ In addition to its role in the proteolytic degradation of Aβ, the levels of SST peptide are downregulated in the early stages of Alzheimer’s disease, especially in the hippocampus and neocortex,^9,10^ areas within the brain from where Aβ pathology initially progresses.^11^ Colocalization of SST and its receptors with Aβ pathology in the brain and its ability to activate neprilysin suggest the use of the peptide as a promising therapeutic option in Alzheimer’s disease.

**Figure 1:**
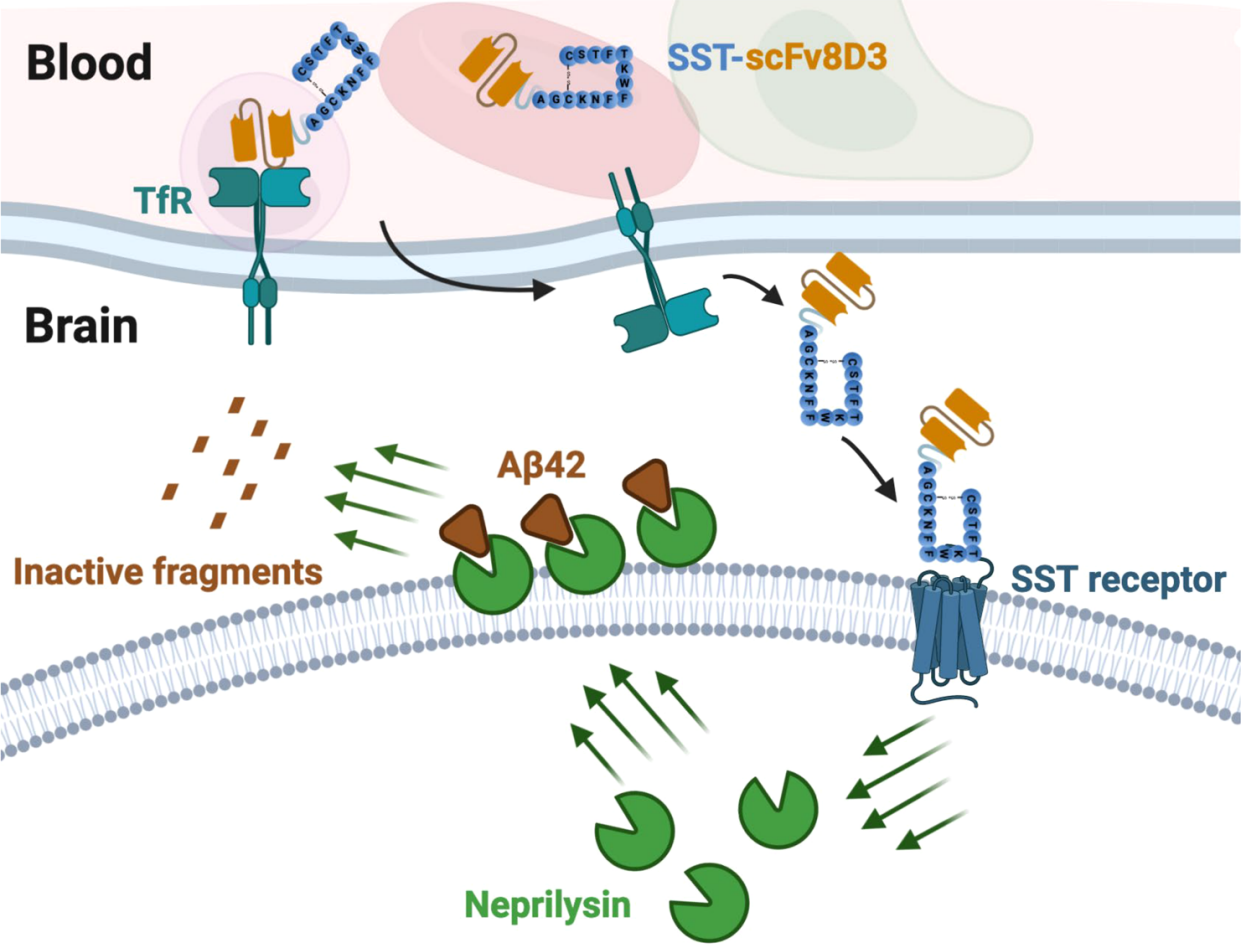
Intravenously injected SST-scFv8D3 passes from blood to brain by binding to the transferrin receptor (TfR). In the brain, SST-scFv8D3 interacts with SST-receptors. This interaction causes a series of downstream events, pushing neprilysin toward the membrane of the neurons, where it can degrade membrane-bound Aβ42 into inactive fragments.

We have previously shown the high brain uptakes and long plasma half-life of the 14 amino acid-long SST peptide recombinantly expressed with a protein-based blood–brain barrier (BBB) transporter when injected intravenously.^6^ The scFv8D3 transporter of the SST peptide binds to the mouse transferrin receptor (TfR), which has previously been used to increase brain uptake of protein-based drugs used in the diagnosis and treatment of Alzheimer’s disease.^12−14^ The recombinantly expressed protein (SST-scFv8D3) increased both the neprilysin concentration and activity within the brain of APPswe mice. In addition, our previous study displayed a significant reduction in the concentration of the membrane-bound aggregation prone Aβ42 in the hippocampal area after triple intravenous injections of 1 mg/kg SST-scFv8D3.^6^ While these effects highlighted SST-scFv8D3 as a potential therapeutic option in the treatment of early Alzheimer’s disease, changes in the levels of other proteins in the brain resulting from such treatment need to be addressed. Regarding binding of SST-scFv8D3 to SSTRs, the subsequent enhancement of neprilysin levels and hippocampal Aβ42 degradation is likely associated with significant changes in the levels of other as of yet unknown proteins involved in Alzheimer’s disease pathology (Figure 1).

In this study, we aimed to integrate anti-Alzheimer’s-disease-related effects from the treatment with SST-scFv8D3 with untargeted analysis of the brain proteome in APPswe mice. In detail, we evaluated protein levels in the hippocampus, the rest of cerebrum, and the cerebellum, following treatment with SST-scFv8D3. Furthermore, a transgenic study of untreated APPswe and wild-type (WT) mice was conducted to evaluate whether SST-scFv8D3 treatment shifts the altered proteins toward wild-type levels or not. Interesting targets identified by the proteomic analysis with LC–MS were further analyzed by Western blot. In addition to the results generated from LC–MS analysis, we aimed to study the effects of such treatment on inflammation, as the latter plays a major role in the pathology of Alzheimer’s disease.^15^ In our previous study, no significant differences were detected in the concentration of interleukins after treatment with SST-scFv8D3.^6^ However, the response of the immune system to such treatment could include processes that leave interleukin levels unaltered. Therefore, a multiplex pro-inflammatory cytokine panel analysis was conducted to quantify the concentration of other inflammatory markers, such as growth-regulated α (KC/GRO) chemokine, in response to SST-scFv8D3 treatment. Finally, brain concentrations of peptide substrates for neprilysin other than Aβ were quantified following treatment with SST-scFv8D3, to evaluate the downstream effects of enhanced neprilysin activation.

## Results

**Figure 2:**
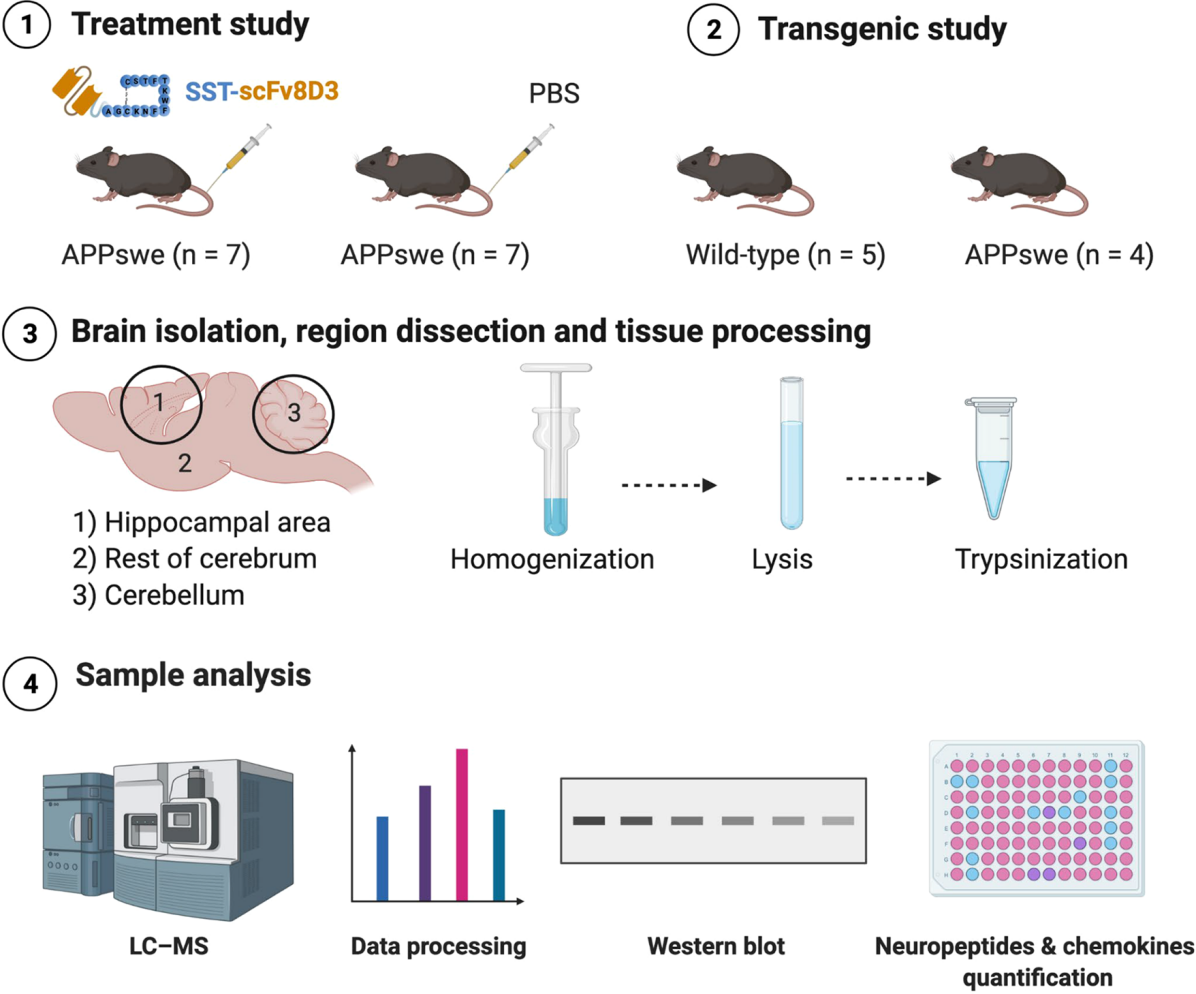
Schematic workflow of the study. For the treatment study, eight-month-old transgenic mice with an Alzheimer’s-like pathology (APPswe) were divided into two groups. The first group (*n* = 7) was injected intravenously with 1 mg/kg SST-scFv8D3 every 36 h (three injections in total). The second group (*n* = 7) was injected intravenously with PBS at the same time points as those of the SST-scFv8D3 group. Mice were euthanized 24 h after the last injection. For the transgenic study, C57Bl/6 wild-type mice (*n* = 5) and APPswe mice (*n* = 4) at the same age as the mice included in the treatment study were used. Brains were isolated and dissected into three parts: hippocampal area, rest of cerebrum, and cerebellum. This was followed by homogenization with Tris buffer, lysis with HEPES/Urea buffer, and trypsinization, followed by analysis with LC–MS, Western blot, and quantification of neuropeptides and chemokines.

To evaluate the effects of SST-scFv8D3 treatment on the brain proteome, two LC–MS studies were conducted. In the first study, we compared the brain proteome of APPswe mice treated with SST-scFv8D3 with that of APPswe mice treated with PBS; hereafter, this is referred to as the treatment study. The second study aimed at comparing the brain proteome of untreated APPswe mice with that of WT controls; hereafter, this is referred to as the transgenic study. A schematic illustration of the workflow can be found in Figure 2.

### LC–MS Analysis of the Hippocampal Area, Rest of Cerebrum, and Cerebellum (Treatment Study)

**Figure 3:**
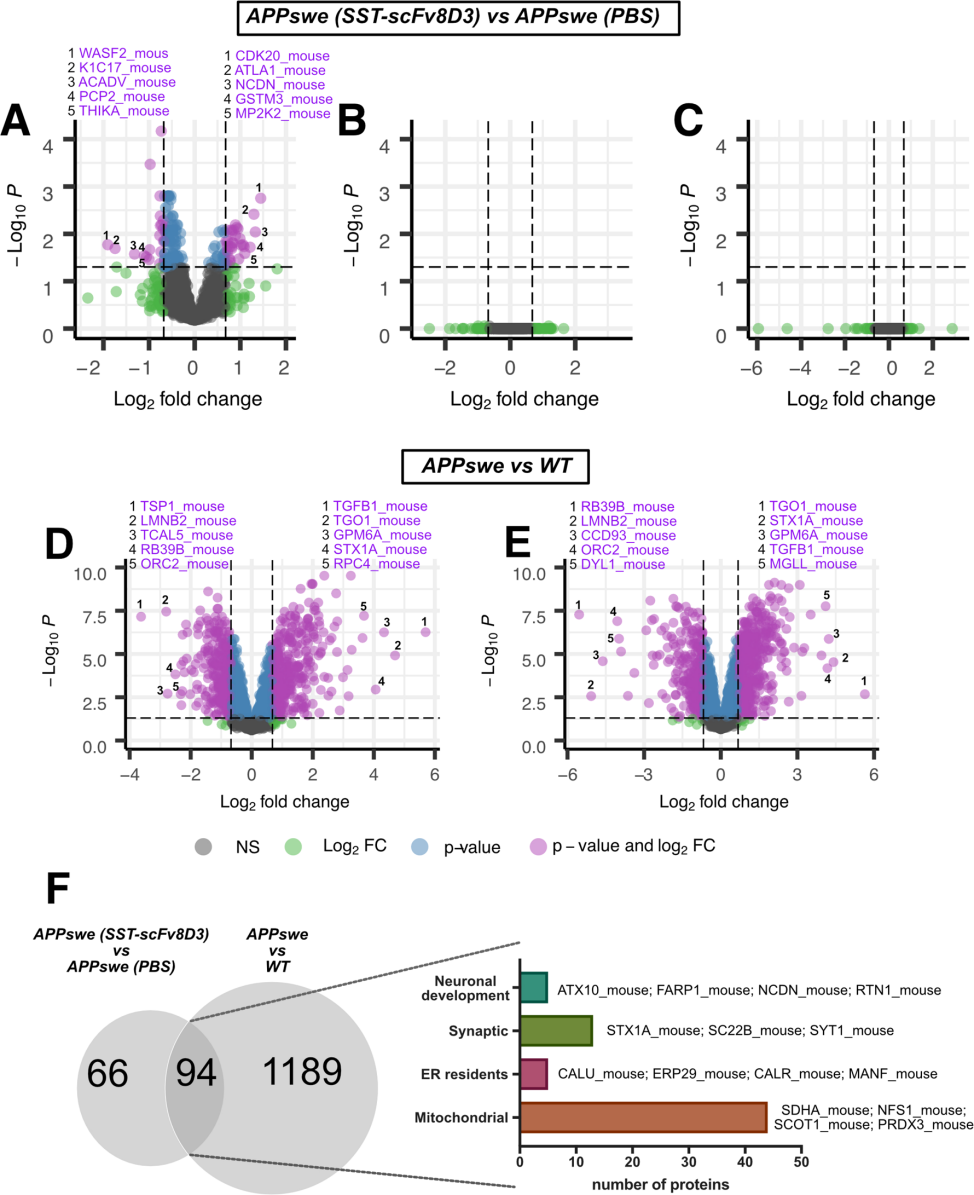
Summary of the LC–MS results. Volcano plots showing (A) hippocampal proteins with different levels between SST-scFv8D3 treated and PBS treated APPswe mice, (B and C) no significant alterations in protein levels between SST-scFv8D3 treated and PBS treated APPswe mice in the rest of cerebrum and cerebellum, (D) hippocampal proteins with different levels between APPswe and wild-type (WT) mice, and (E) proteins in the rest of cerebrum with different levels between APPswe and WT mice. Significantly affected proteins (*p* < 0.05) are displayed in blue and purple; not significantly (NS) affected proteins are presented in gray and green, whereas purple and green indicate a |log_2_(Fold change)| > log_2_(1.6). The top five significantly altered proteins based on fold change are highlighted in purple. (F) Overlap of all differentially expressed hippocampal proteins between SST-scFv8D3 treated and PBS treated APPswe mice with untreated APPswe and WT mice. Examples from the 94 proteins that overlapped between the two studies are presented. The majority of these proteins (44 out of 94) are mitochondrial.

The proteome of the hippocampus, rest of the cerebrum, and cerebellum, from seven SST-scFv8D3 treated and seven PBS treated transgenic APPswe mice, were analyzed with LC–MS. In total, 1869 proteins could be quantified across the 42 samples (Table S1). In the hippocampal area, the levels of 55 proteins were significantly increased, and 105 proteins were significantly decreased in the SST-scFv8D3 treated APPswe mice compared to those of the PBS injected group (Figure 3A). No gender-specific differences were detected between SST-scFv8D3 treated and PBS treated APPswe mice.

No significant differences were found in either the cerebellum or in the rest of the cerebrum (Figure 3B,C), which is in line with our previous observations.^6^ Mitochondrial and endoplasmic reticulum resident proteins represented the majority of proteins with significantly lower levels in the hippocampus of SST-scFv8D3 treated APPswe mice (Table S2 and Figure S1). Synaptic proteins involved in regulating trafficking across the cell membrane and neuronal development represented the majority of the proteins with significantly higher levels in the hippocampus of SST-scFv8D3 treated APPswe mice (Table S3 and Figure S1). The top 10 biological processes enriched due to changes in hippocampal protein levels of APPswe mice after treatment with SST-scFv8D3 are illustrated in Table 1. In addition, the altered proteins were mainly associated with mitochondria as displayed in the top 10 cellular components in Table 2.

**Table 1:** Gene Ontology (GO) Enrichment Analysis Showing the Top 10 GO Terms for Biological Processes^a^

GO biological process	detected	expected	fold enrichment	raw *p*-value	FDR
fatty acid β-oxidation using acyl-CoA dehydrogenase	6	0.57	10.55	1.80 × 10^–04^	4.95 × 10^–02^
tricarboxylic acid cycle	14	1.54	9.07	2.44 × 10^–08^	7.15 × 10^–05^
branched-chain amino acid catabolic process	7	0.81	8.62	1.21 × 10^–04^	3.80 × 10^–02^
branched-chain amino acid metabolic process	7	0.81	8.62	1.21 × 10^–04^	3.67 × 10^–02^
acetyl-CoA metabolic process	7	0.89	7.84	1.86 × 10^–04^	4.95 × 10^–02^
aerobic respiration	15	1.95	7.70	3.72 × 10^–08^	8.16 × 10^–05^
cellular respiration	18	2.52	7.15	3.44 × 10^–09^	3.02 × 10^–05^
purine nucleoside bisphosphate metabolic process	12	1.87	6.42	3.93 × 10^–06^	2.87 × 10^–03^
ribonucleoside bisphosphate metabolic process	12	1.87	6.42	3.93 × 10^–06^	2.65 × 10^–03^
nucleoside bisphosphate metabolic process	12	1.87	6.42	3.93 × 10^–06^	2.46 × 10^–03^

a Processes are of the significantly altered proteins in the hippocampal area of APPswe mice following SST-scFv8D3 treatment.

**Table 2:** Gene Ontology Enrichment Analysis Showing the Top 10 GO Terms for Cellular Components^a^

GO cellular component	detected	expected	fold enrichment	raw *p*-value	FDR
tricarboxylic acid cycle enzyme complex	6	0.57	10.55	1.80 × 10^–04^	1.66 × 10^–02^
oxidoreductase complex	10	1.46	6.84	1.74 × 10^–05^	2.31 × 10^–03^
mitochondrial matrix	26	5.28	4.92	5.05 × 10^–10^	3.02 × 10^–07^
mitochondrial inner membrane	17	4.87	3.49	3.10 × 10^–05^	3.71 × 10^–03^
organelle inner membrane	17	5.04	3.38	4.42 × 10^–05^	4.40 × 10^–03^
mitochondrial membrane	23	7.23	3.18	4.12 × 10^–06^	6.15 × 10^–04^
mitochondrial envelope	26	8.20	3.17	9.43 × 10^–07^	2.81 × 10^–04^
envelope	34	13.16	2.58	1.14 × 10^–06^	2.72 × 10^–04^
organelle envelope	34	13.16	2.58	1.14 × 10^–06^	2.27 × 10^–04^
mitochondrion	74	30.78	2.40	2.35 × 10^–13^	2.80 × 10^–10^

a Components are of the significantly altered proteins in the hippocampal area of APPswe mice following SST-scFv8D3 treatment.

### LC–MS Analysis of the Hippocampal Area and Rest of Cerebrum (Transgenic Study)

In order to evaluate whether SST-scFv8D3 treatment shifts the altered proteins toward WT levels, untreated APPswe and WT mice of the same age as the above-mentioned APPswe treated groups were compared in the transgenic study. Hippocampus and rest of cerebrum from four APPswe and five WT mice were analyzed with LC–MS as described above (Figure 2). In total, 2487 proteins could be detected across the 18 samples (Table S4). In the hippocampal area, the levels of 1283 proteins were significantly altered (Figure 3D). In the rest of the cerebrum, the levels of 1379 proteins were significantly altered (Figure 3E). Out of these, 922 proteins were significantly altered in both regions. Amyloid-β precursor protein (A4_mouse) was among the proteins with significantly increased levels in APPswe compared to those of WT mice in both regions (Table S4). Other proteins like the synaptic protein syntaxin-1A (STX1A_mouse) and the transforming growth factor β-1 (TGFB1) protein (TGFB1_mouse) represented some of the proteins with the most significant increase in the brains of APPswe mice compared to WT mice (Figure 3D,E). Proteins involved in autophagy such as the Ras-related proteins (Rab39) (RB39B_mouse) and the nuclear protein Lamin-B1 (LMNB1_mouse) represented some of the proteins with the most significantly decreased levels in the brain of APPswe mice compared to WT mice (Figure 3D,E).

Of the 160 differentially expressed proteins between SST-scFv8D3 and PBS treated APPswe, 94 proteins overlapped with the 1283 differentially expressed proteins between APPswe and WT mice in the hippocampus (Figure 3F and Table S5). Mitochondrial proteins represented the majority of these proteins (44 out of 94), followed by synaptic proteins, ER-resident proteins, and proteins involved in neuronal development (Figure 3F). Out of these 94 proteins, treatment with SST-scFv8D3 shifted the levels of 64 proteins toward WT levels (Table S5).

### Syntaxin-1A Quantification

**Figure 4:**
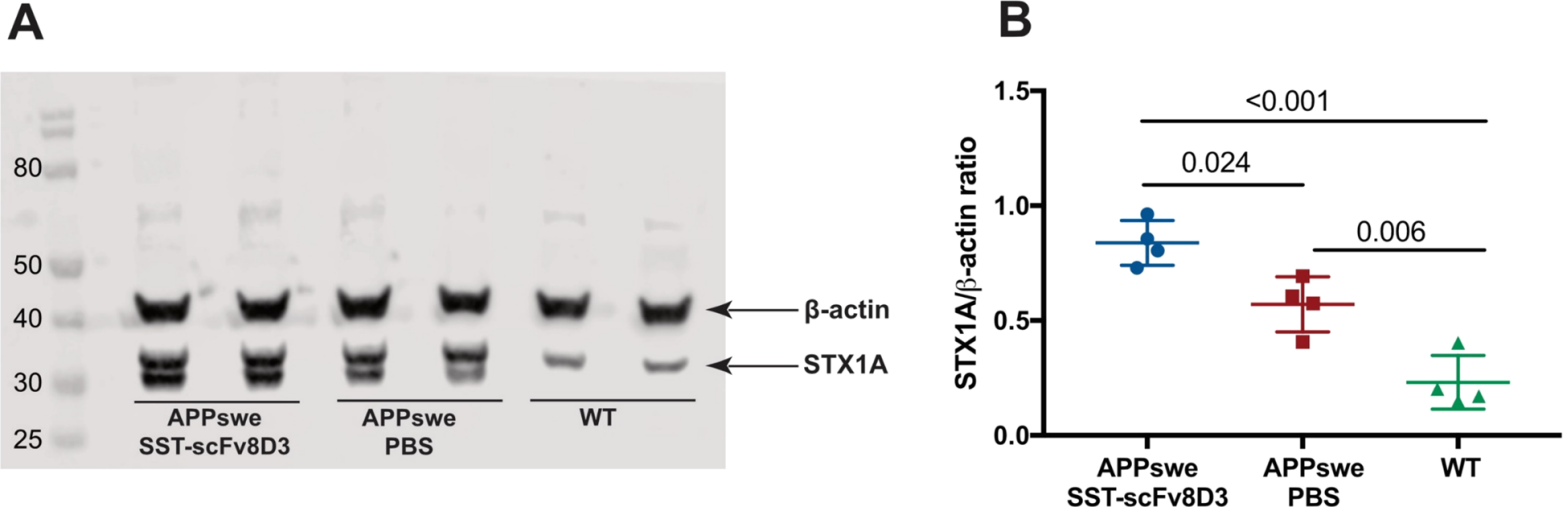
Western blot analysis of hippocampal lysates from SST-scFv8D3 treated APPswe mice (*n* = 4), PBS treated APPswe (*n* = 4), and WT controls (*n* = 4) for the detection and quantification of syntaxin-1A. (A) Representative Western blot image displaying qualitatively higher signals of syntaxin-1A in the SST-scFv8D3 treated APPswe lysates compared to those of PBS treated ones. Higher signals of syntaxin-1A in APPswe groups compared to those of WT controls. (B) Normalization against β-actin revealed a statistically significant increase in synatxin-1A in the hippocampal area of SST-scFv8D3 treated group compared to that of PBS treated APPswe mice. A statistically significant increase in synatxin-1A detected in the hippocampal area of APPswe mice compared to that of WT controls. Results are presented as mean ± SD. One-way ANOVA followed by Bonferroni’s posthoc test was applied to measure the presence of statistically significant differences in the results. A significant *p*-value is defined as *p* < 0.05.

As mentioned in the previous section, synaptic proteins regulating trafficking across cell membranes represented most of the proteins with increased levels after treatment with SST-scFv8D3. Synaptic dysfunction is one of the main pathological events in Alzheimer’s disease, where the interaction between Aβ and syntaxin-1A has been demonstrated as a major contributing factor.^16^ The levels of the latter protein were increased in the hippocampal area of APPswe after treatment with SST-scFv8D3 measured with LC–MS (adjusted *p*-value 0.007) (Table S3). Moreover, syntaxin-1A was the fourth most significantly increased protein in the hippocampus of APPswe mice compared to WT controls (adjusted *p*-value < 0.0001) (Table S4). To confirm these results, semiquantitative Western blot analysis was performed. Lysates from the hippocampal area of SST-scFv8D3 treated APPswe mice displayed a significant increase in syntaxin-1A compared to that of the PBS treated group (Figure 4). In addition, APPswe mice displayed a significant increase in syntaxin-1A compared to that of the WT controls (Figure 4), further confirming the LC–MS results.

### KC/GRO Chemokine Quantification

**Figure 5:**
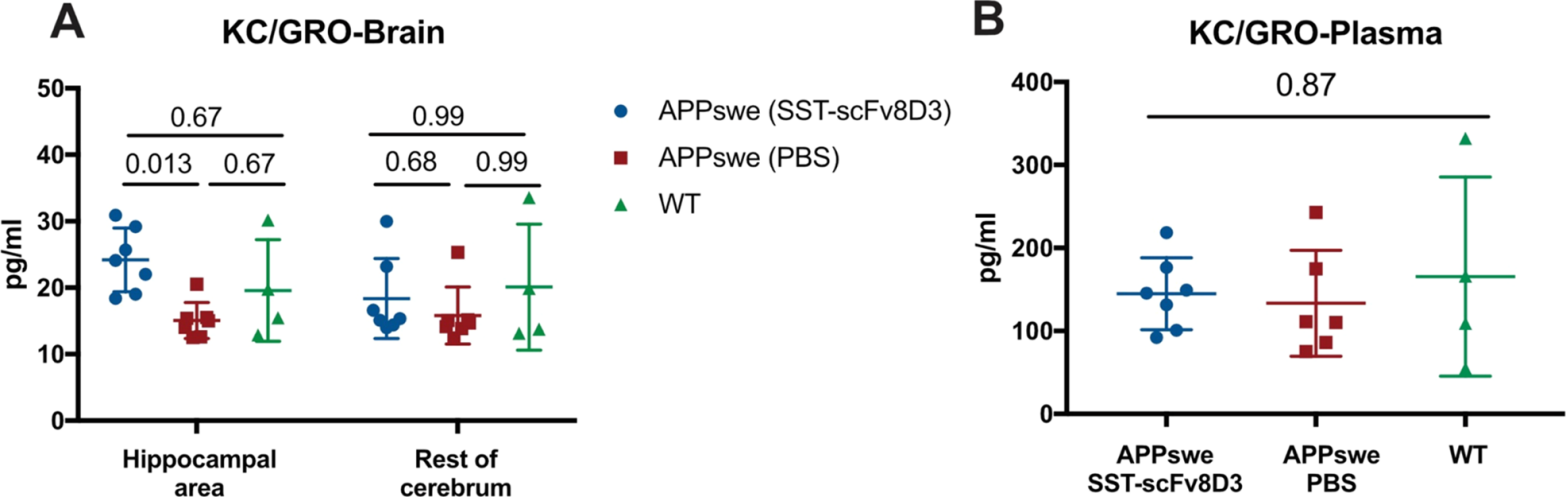
Concentration of KC/GRO in the brain and blood of eight-month-old APPswe mice after triple intravenous injection of 1 mg/kg SST-scFv8D3 or PBS, using age-matched WT mice as controls. (A) A significant increase in hippocampal concentrations of KC/GRO in the SST-scFv8D3 treated group compared to those of PBS treated APPswe mice. No differences detected between the groups in the rest of the cerebrum (*n* = 7 per group for APPswe mice) (*n* = 4 for WT mice). (B) No significant differences detected in plasma concentration of KC/GRO between SST-scFv8D3 treated APPswe (*n* = 7), PBS treated APPswe (*n* = 6), and WT mice (*n* = 4). Quantifications performed using a pro-inflammatory multiplex assay (MSD K125QTD). Results are presented as mean ± SD. Kruskal–Wallis test followed by Dunn’s post hoc analysis was applied to measure the presence of statistically significant differences in the results. A significant *p*-value is defined as *p* < 0.05.

In our previous study, no significant differences were detected in the concentration of interleukins (IL-1b, IL-5, IL-6, and IL-12) after treatment with SST-scFv8D3.^6^ However, the response of the immune system to such treatment could include changes in the levels of inflammatory markers other than interleukins. In the current study, we wanted to study immune cell activation and migration. For this purpose, we quantified the concentration of growth-regulated α (KC/GRO), a chemokine that regulates the activation of neutrophils and macrophages.^17^ This chemokine was not detected in our LC–MS analysis. Using the MSD multiplex assay, a significant increase in the concentration of KC/GRO was detected in the hippocampal area of SST-scFv8D3 treated APPswe mice compared to that of the PBS treated group (Figure 5A). No differences were detected in the rest of the cerebrum (Figure 5A). The increased concentrations of KC/GRO were hippocampal-specific, as no differences were detected in the concentration of this chemokine in the blood (Figure 5B).

### Neuropeptide-Y and Substance-P Quantification

**Figure 6:**
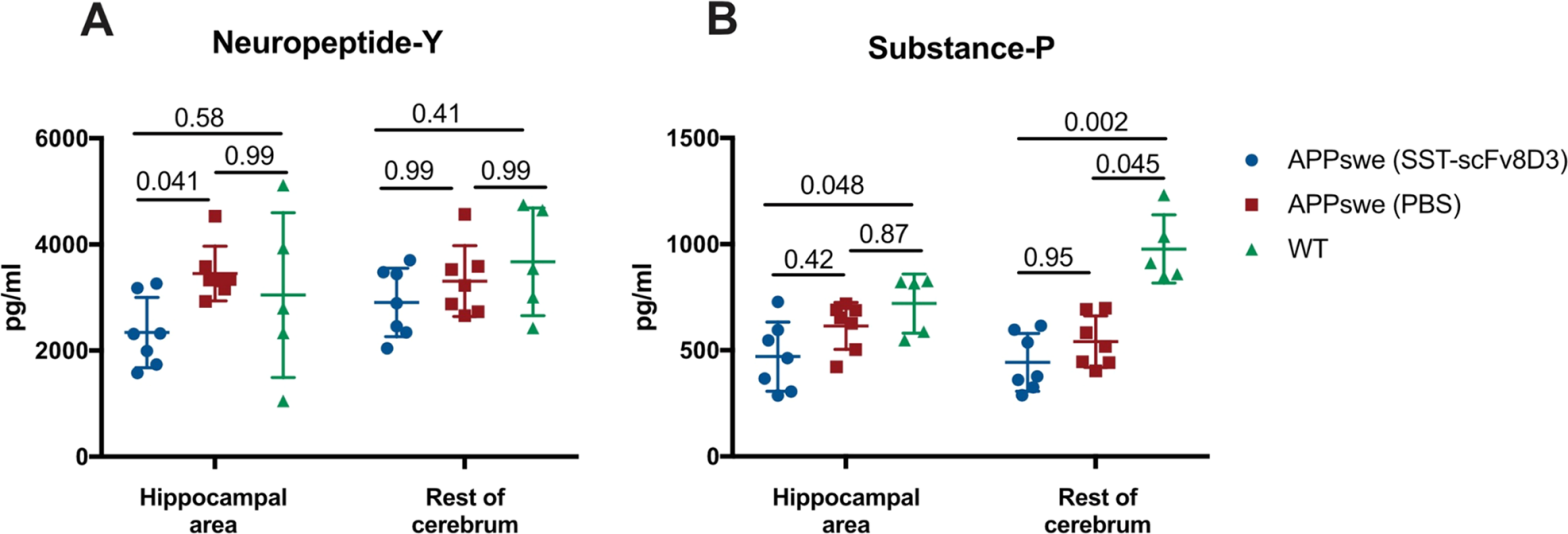
Brain concentrations of neuropeptide-Y and substance-P in eight-month-old APPswe mice after triple intravenous injection of 1 mg/kg SST-scFv8D3 or PBS, using age-matched WT mice as controls. (A) A significant decrease in hippocampal concentrations of neuropeptide-Y in the SST-scFv8D3 treated APPswe compared to those of the PBS treated APPswe mice. No differences detected between the groups in the rest of cerebrum. (B) No significant differences detected in the concentration of substance-P between SST-scFv8D3 treated and PBS treated APPswe mice. A significant decrease in substance-P concentration in APPswe mice compared to WT controls. Results are presented as mean ± SD. Kruskal–Wallis test followed by Dunn’s post hoc analysis was applied to measure the presence of statistically significant differences in the results. A significant *p*-value is defined as *p* < 0.05. (*n* = 7 for APPswe treated with SST-scFv8D3; *n* = 7 for APPswe treated with PBS; *n* = 5 for WT).

We have previously demonstrated the ability of the bispecific SST-scFv8D3 protein drug to degrade hippocampal Aβ42 as a result of enhanced neprilysin activation.^6^ In addition to Aβ, neprilysin degrades other peptides in the brain, including neuropeptide-Y and tachykinins such as substance-P.^18^ In the current study, we aimed at quantifying the brain concentration of other neprilysin substrates, focusing on neuropeptide-Y and substance-P. A significant decrease in the concentration of neuropeptide-Y was detected only in the hippocampal area of the SST-scFv8D3 treated group (Figure 6A), the area where neprilysin activity was selectively enhanced.^6^ No differences were detected in the brain concentration of substance-P between SST-scFv8D3 treated and PBS treated APPswe mice (Figure 6B). APPswe mice demonstrated a significant decrease in substance-P concentration compared to that of the WT controls (Figure 6B).

## Discussion

In this study, we demonstrate the ability of the SST peptide with a protein-based BBB transporter (SST-scFv8D3) to regulate the levels of several proteins in the hippocampus of the APPswe transgenic mouse model of Alzheimer’s disease. The effects were selectively targeted to the hippocampal area of the brain, where SST receptors are highly expressed. We have previously displayed the ability of SST-scFv8D3 to enter the brain parenchyma and to localize predominately in the hippocampus and its surrounding cortical area, selectively enhancing neprilysin expression and reducing the Aβ42 concentration in this brain region.^6^ No effects of the formed fusion protein on the proteome of the cerebellum or rest of the cerebrum were observed.

The altered protein levels measured in this study after treatment with SST-scFv8D3 can be attributed to several possible reasons. The removal of excess Aβ peptides could be followed by the alteration in hippocampal protein levels detected in the SST-scFv8D3 treated group. However, the observed changes could also be related to SST receptor activation that is associated with numerous downstream events unrelated to neprilysin activation. Furthermore, neprilysin-mediated degradation of substrates other than Aβ, and the interaction of SST with other proteins, could also contribute to the detected effects in this study.

Because our protein design also includes a BBB transporter, it is possible that some of the effects are mediated by this part of the construct rather than the SST. The scFv8D3 transporter binds to TfR and induces endocytosis of this complex. TfR is abundantly expressed on the endothelial cells of the BBB but not on other cell types in the brain.^19^ The analyzed brain regions in this study contain a small proportion of BBB cells, resulting in a possible alteration of protein levels related to the enhanced endocytosis or other downstream effects due to the interaction with TfR. Nevertheless, we have previously demonstrated an even brain distribution of the BBB transporter, which is not limited to a specific region.^14^ However, the SST-scFv8D3 construct has a longer retention time in the hippocampal area, where the SST receptors are highly expressed,^6^ indicating that TfR binding is not as essential for half-life extension as the interaction with SST receptors. Because the observed changes in protein levels were exclusive to the hippocampal area (Figure 3A), SST-scFv8D3 interaction with TfR is only likely to contribute to a small proportion of the detected alterations in protein levels after treatment. Having this in mind, the following sections are based on discussing the altered protein levels only in regard to Alzheimer’s disease:

### Proteins with the Most Significant Decrease (Based on Fold Change) after SST-scFv8D3 Treatment

The top 10 proteins whose levels were significantly decreased after treatment with SST-scFv8D3 were mainly mitochondrial and endoplasmic reticulum (ER) residents (6 out of 10), which are further described in the following sections. Two plasma membrane proteins were also detected among the top 10 downregulated proteins after SST-scFv8D3 treatment. These included Wiskott-Aldrich syndrome protein family members 2 (highest significant decrease in terms of fold-change after treatment with SST-scFv8D3), which is involved in the processes of protein folding and Rho GTPase-activating protein 35, which is involved in cell differentiation. In addition, the two extracellular proteins Clathrin light chain B and Purkinje cell protein 2 represented the rest of the top 10 proteins with the most significant decrease. The latter protein, Purkinje cell protein 2, is involved in modulating cell-signaling processes. In the following sections, we discuss interesting leads from the downregulated proteins in relation to their involvement in Alzheimer’s disease pathology.

#### Mitochondrial Proteins

In our treatment study, around half of the proteins whose levels were significantly decreased in the SST-scFv8D3 treated group were mitochondrial, involved mainly in processes of fatty acid oxidation, amino acid metabolism, energy production, autophagy, and protein assembly. Mitochondrial dysfunction is one of the key pathological events contributing to the onset and progression of Alzheimer’s disease.^20^ Several studies have demonstrated the ability of aggregated Aβ to accumulate and interact with mitochondrial proteins, both in transgenic mouse models overexpressing Aβ and in Alzheimer’s disease patients’ brains.^21,22^ Transgenic Alzheimer’s disease mice harboring the Swedish mutation exhibited mitochondrial accumulation of Aβ40 and 42 isoforms at early stages of the disease before plaque onset.^23^ The proposed mechanisms for Aβ-induced mitochondrial dysfunction include decline in the mitochondrial import of physiologically relevant proteins, increased reactive-oxygen species (ROS), and decreased mitochondrial membrane potential.^24^ To compensate for such effects, several mitochondrial proteins are upregulated in the early stages of the disease. This upregulation could be attributed to proteins that propagate the disease or proteins that contribute to a compensatory mechanism that protects the mice from the overexpression of Aβ and the subsequent downstream effects. Examples include thioredoxin-reductase and citrate synthase that have been previously shown to be upregulated in Alzheimer’s disease brains^25^ and in an 8-month-old transgenic mouse model of Alzheimer’s disease,^26^ respectively. These two mitochondrial proteins among several others were downregulated in the hippocampus of the study group, which could be a sign of an altered mitochondrial function resulting from enhanced degradation of Aβ42 by SST-scFv8D3. In order to assess whether the effects of SST-scFv8D3 treatment re-establishes normal hippocampal levels of the mitochondrial proteins, we performed a transgenic study including age-matched APPswe mice and WT controls. Interestingly, 80% of the mitochondrial proteins that were downregulated after SST-scFv8D3 treatment displayed significantly higher levels in the hippocampi of APPswe mice compared to those of WT mice, strongly suggesting that SST-scFv8D3 corrects these altered mitochondrial proteins toward WT levels. The data obtained in the transgenic study are also consistent with previously published findings, which show a significant increase in the levels of mitochondrial proteins including stress-70 protein, 10 kDa heat shock protein, superoxide dismutase, fumarate hydratase, aspartate aminotransferase, and pyruvate dehydrogenase in the brains of neprilysin knockout APPswe mice compared to those of neprilysin knockout WT mice.^25^ In our study, all these mentioned proteins are downregulated in the hippocampi of APPswe mice treated with SST-scFv8D3, an effect that could be mediated by the enhanced degradation of Aβ42. These results could indicate that the observed increase in the levels of the mitochondrial proteins was independent of neprilysin levels and instead was due to Aβ overproduction and aggregation. However, SST peptide has displayed the ability of binding and interacting with several mitochondrial proteins, including aldehyde dehydrogenase in brain homogenates,^27^ which was also downregulated in our study. Moreover, neprilysin inhibitors used in the treatment of heart failure has been associated with an increase in the levels of mitochondrial proteins,^28^ several of which are downregulated in our study where neprilysin levels are enhanced.^6^ These studies might suggest that the regulation of mitochondrial proteins is not exclusively mediated by decreased Aβ levels but can also be mediated by SST interaction with other proteins and neprilysin overexpression.

#### Endoplasmic Reticulum Proteins

In addition to mitochondria, several of the proteins with significantly lower levels in the study group are ER residents. The expression and function of these proteins in Alzheimer’s disease are not well-studied. However, the ER can provide a suitable oxidizing environment for the processing of APP and generation of Aβ peptides, where ER-resident proteins such as calreticulin play a role.^29^ In the current study, the levels of calreticulin as well as other ER proteins such as calumenin and endoplasmic reticulum resident protein 29 were significantly decreased in the hippocampi of APPswe mice compared to those of the WT controls. Treatment of APPswe mice with SST-scFv8D3 decreased their levels even more, an effect that needs further investigation.

#### Amyloid-Like Protein 1 (APLP-1)

Importantly, treatment with SST-scFv8D3 was associated with a 40% decrease in the hippocampal levels of amyloid-like protein 1 (APLP-1). No studies have demonstrated the ability of neprilysin to degrade APLP-1. However, interaction between the latter protein and neprilysin promoter has been previously reported.^30^ The involvement of APLP-1 in Alzheimer’s disease has been extensively discussed. APLP-1 is a member of the amyloid precursor family and is highly homologous to APP, showing similar enzymatic digestion processing into short Aβ-like peptides that accumulate in Alzheimer’s disease brains.^31,32^ Moreover, APLP-1 has been suggested as a cerebrospinal fluid (CSF) biomarker in Alzheimer’s disease.^33^ Decreased levels of this protein in the hippocampal area might further support the anti-Alzheimer’s effects of SST-scFv8D3.

#### Neuropeptide-Y and Substance-P

We have previously demonstrated a significant decrease in the concentration of Aβ42 in the hippocampi of APPswe mice after SST-scFv8D3 treatment.^6^ This is an effect that was mediated by the significant and selective increase in hippocampal neprilysin activity.^6^ However, neprilysin is a promiscuous enzyme capable of degrading a variety of substrates in the brain other than Aβ.^18^ For this reason, we quantified the brain concentrations of neuropeptide-Y and substance-P, two peptide substrates of neprilysin that are highly expressed in the brain. Treatment with SST-scFv8D3 was associated with a significant decrease in neuropeptide-Y concentration, but not substance-P, selectively in the hippocampal area (Figure 6). Enhanced degradation of neuropeptide-Y in the hippocampus could further demonstrate the increased activity and expression of neprilysin in this specific brain region after SST-scFv8D3 treatment. However, the enhanced degradation of neuropeptide-Y might be associated with possible adverse effects, because the peptide is involved in regulating several relevant physiological processes in the brain such as food intake and energy homeostasis. Of note, APPswe mice demonstrated a significant decrease in substance-P concentration compared to that of WT controls, an effect that was even more evident in areas outside the hippocampus as demonstrated previously (Figure 6).^34^

### Proteins with the Most Significant Increase after SST-scFv8D3 Treatment

Proteins regulating trafficking across the cell membrane represented around 40% of the proteins with significantly higher levels found in the hippocampal area of SST-scFv8D3 treated APPswe mice. Proteins involved in processes of axon and dendrite assembly, synapse formation, cell-to-cell adhesion, and ubiquitination represented the rest of the upregulated proteins. In the following sections, we discussed some of these proteins that are involved in Alzheimer’s disease pathology.

#### KC/GRO Chemokine

Inflammation plays a major role in the pathology of Alzheimer’s disease.^15^ We have previously demonstrated no significant alteration in the concentrations of interleukins (IL-1b, IL-5, IL-6, and IL12) in the brains of APPswe mice after treatment with SST-scFv8D3.^6^ However, this is not a complete list of cytokines, and the immune system might respond to treatments in several ways that do not include alterations in interleukin concentrations. To further study immune cell-activation, we quantified the concentration of KC/GRO, a chemokine expressed in endothelial cells and macrophages. This chemokine plays an integral role in the activation and migration of immune cells like neutrophils.^17^ Its receptor, CXCR2, is expressed by neurons and microglia.^35^ Treatment with SST-scFv8D3 was associated with a significant and selective increase in KC/GRO concentration in the hippocampal area (Figure 5A). No changes were detected in the rest of the cerebrum, nor in the blood, indicating that the increase in KC/GRO concentration is correlated with SST-scFv8D3 treatment effects. Downstream effects of increased KC/GRO concentration by SST-scFv8D3 treatment could contribute to pro-inflammatory microglial activation mediated by the interaction of KC/GRO with its receptor.^36^ In addition, enhanced expression of KC/GRO can be associated with infiltration of immune cells from the periphery into the brain, including both monocytes^37^ and neutrophils,^38^ aiding the phagocytic removal of degraded Aβ. Furthermore, our results could hint to the important effects that KC/GRO chemokine might exert in the early stages of the disease.

#### Syntaxin-1A

An interesting protein that was upregulated in the hippocampus of SST-scFv8D3 treated APPswe mice was the SNARE (soluble *N*-ethylmaleimide-sensitive fusion protein attachment protein receptor) protein syntaxin-1A. The enhanced levels of this protein in the SST-scFv8D3 treated group were also confirmed with Western blot (Figure 4). Furthermore, syntaxin-1A was among the most significantly upregulated proteins in the brain of APPswe mice compared to WT controls (Figure 3D,E). Although previous studies have demonstrated reduced syntaxin-1A levels in Alzheimer’s disease brains,^39^ the observed increase in the levels of this protein in our transgenic mouse model can be a compensatory mechanism to the overexpression of APP in the brain of these mice. Importantly, Aβ oligomers have shown the ability of binding to syntaxin-1A, inhibiting SNARE complex formation and subsequently reducing SNARE-mediated vesicle fusion.^16^ Treatment of APPswe mice with SST-scFv8D3 further increased syntaxin-1A levels (Figures 3A and 4). This can be attributed to the role of SST in regulating the presynaptic localization of neprilysin, which has been suggested to be partially mediated by SNARE proteins, including syntaxin-1A.^8^

#### Other SNARE-Related Proteins

In addition to syntaxin-1A, the levels of other SNARE-related proteins such as synaptotagmin-1 and synaptobrevin homologue YKT6 were also increased in the hippocampi of SST-scFv8D3 treated mice. Although no interaction between Aβ and these proteins has been reported, their involvement in Alzheimer’s disease has been previously discussed. Synaptotagmin has been shown to have the ability to promote Aβ generation.^40^ In addition, our current transgenic study further demonstrated a significant increase in hippocampal levels of synaptotagmin in APPswe mice compared to those of WT controls. However, knockdown of neprilysin in airway epithelial cells has been associated with a significant decrease in synaptotagmin gene expression.^41^ With that said, it is rather likely that the increased synaptotagmin levels we see in the hippocampi are due to the increase in the neprilysin concentration.

#### Neuronal Growth Proteins

Treatment with SST-scFv8D3 displayed a significant increase in the levels of proteins involved in neuronal growth and development. Examples include serine/threonine protein kinase BRSK2 (highest significant increase in terms of fold-change after treatment with SST-scFv8D3), atlastin-1 (second highest significant increase after treatment), and ataxin-10. The latter protein has recently been suggested to be related to Alzheimer’s disease, as Aβ accumulation and neuronal degeneration were observed in the hippocampus when ataxin-1 is knocked out in a transgenic mouse model of Alzheimer’s disease.^42^ Serine/threonine protein phosphatase 2A (gamma and epsilon isoforms) was also among the upregulated proteins in the treatment group. Overproduction and accumulation of Aβ caused a strong downregulation of the activity of this enzyme in the brain during Alzheimer’s disease.^43,44^ In addition, coimmunoprecipitation studies showed the interaction between neprilysin and serine phosphatase, as well as treatment of primary cultures with serine-phosphatase inhibitors, significantly reduced cell-surface neprilysin activity.^45^ Therefore, enhanced neprilysin activity and reduced Aβ accumulation by our SST-scFv8D3 could probably explain the observed upregulated levels of this enzyme. Finally, another interesting protein group whose levels are significantly increased after treatment is the reticulons (reticulons 1, 3, and 4). In addition to their role in mediating trafficking across the cell membrane, they are involved in neuronal and glial cell growth. They have been involved in modulating the activity of β-secretase, the enzyme involved in the proteolytic digestion of amyloid-precursor protein (APP) into Aβ. Enhancing the expression levels of these proteins has been associated with reduced accumulation of Aβ.^46^ Importantly, our current study demonstrated a significant decrease in the levels of neuronal growth proteins (ataxin-10, neurochondrin, and reticulon-1, among others) in the hippocampi of APPswe mice compared to those of WT controls, further suggesting that SST-scFv8D3 treatment shifts these altered proteins toward normal levels.

#### Dynamin-1

It is worth mentioning that treatment with SST-scFv8D3 was associated with increased levels of the Aβ aggregation regulating the protein dynamin-1. This protein is extensively studied in Alzheimer’s disease, where it has been demonstrated to be involved in regulating Aβ formation and aiding its aggregation.^47,48^ Moreover, APPswe mice with neprilysin knockout displayed a significant increase in dynamin-1 levels.^49^ Whether such an unexpected increase is mediated by our Aβ degrading SST-scFv8D3 protein drug or it is secondary to other interaction partners of SST needs to be further investigated. Importantly, despite its role in enhancing Aβ formation, dynamin-1 has demonstrated some neuroprotective role in synapse development in the central nervous system.^47^ Of note, dynamin-1 is involved in mediating endocytosis and synapse development.^50^ Therefore, the increased hippocampal levels of this protein after SST-scFv8D3 treatment might indicate enhanced endocytosis due to scFv8D3 interaction with TfR and/or SST interaction with SST receptors.

#### Dual-Specificity Mitogen Activated Protein Kinases

Another important protein group with significantly increased levels in the treated APPswe mice were the dual-specificity mitogen activated protein kinases (subtype 2 and 1 representing the fifth and sixth most significantly upregulated proteins after SST-scFv8D3 treatment, respectively). These proteins are an integral part of the mitogen-activated protein (MAP) signaling cascade, involved in regulating immune responses and inflammation and promoting cell survival.^51^ They have been suggested as neuroprotective proteins in several brain diseases through their ability to dephosphorylate important MAP-kinases.^52^ In addition, dephosphorylation of APP by these enzymes has been shown to be associated with the decreased ability of β-secretase to cleave APP and produce Aβ.^53^ The significant decrease in the levels of these proteins in the brains of APPswe mice compared to those of WT controls and their upregulation after SST-scFv8D3 treatment might further support the therapeutic significance of our protein-drug.

### Conclusion

We have shown that treatment with SST-scFv8D3 generated multiple anti-Alzheimer’s disease effects. Identification of proteins, such as syntaxin-1A and the chemokine KC/GRO, highlights their potential role in Alzheimer’s disease, opening up new possibilities to further study their association with Aβ pathology. We also saw that mitochondrial and neuronal developmental proteins that are frequently altered in Alzheimer’s disease were shifted by the SST-scFv8D3 treatment towards normal levels. Our findings suggest that the somatostatin peptide not only lowers the amount of Aβ in the hippocampus, as we have shown earlier,^6^ but is also associated with other previously undiscovered beneficial effects. As these effects are closely related to cognition, we believe that the results of this study have strengthened the continued evaluation of the somatostatin peptide as a therapeutic option to ameliorate the cognitive dysfunction presented in Alzheimer’s disease.

## Materials and Methods

### Animals

Female and male 8-month-old APPswe transgenic mice, harboring the Swedish mutation (AβPP KM670/671NL), were used in this study. Littermates were used as wild-type controls. The mice were housed in an animal facility at Uppsala University, with free access to water and food and in rooms with controlled temperature and humidity. Experimental procedures were approved by the Uppsala County Animal Ethics Board (#5.8.18-13350/17).

### SST-scFv8D3 Treatment

APPswe mice were divided into two groups (*n* = 7 per group). Using the tail vein, 1 mg/kg SST-scFv8D3, recombinantly produced as described previously,^6^ was intravenously injected into the study group every 36 h (three injections in total). SST-scFv8D3 is a fusion protein consisting of the 14 amino acid SST peptide (AGCKNFFWKTFTSC) linked to scFv8D3 with an in-house designed linker (APGSYTGSAPG). The second group received an intravenous injection of phosphate buffered saline (PBS) at the same time points as the study group. At 24 h after the last injection, mice were euthanized by transcardial perfusion with 0.9% physiological saline. Brains were isolated, and three brain regions (hippocampal area, rest of cerebrum, and cerebellum) were dissected on ice and stored in −80 °C. The three brain regions were homogenized at a 1:5 weight/volume ratio with the homogenization buffer (20 mM Tris, 137 mM NaCl, pH 7.6) containing 1% (volume/volume) protease inhibitor (Thermo Scientific, Waltham, MA, USA). The same procedures regarding mice euthanization and tissue processing were applied for the transgenic study, including untreated APPswe mice and WT controls.

### Tryptic Digestion

The homogenates of the three brain regions were lysed using tip sonication at 4 °C (pulse 10 × 1 s, rest 1 s, amplitude 30%; Vibra cell ultrasonic processor with 3 mm probe; Sonics, Newtown, CT, USA) in a buffer containing 20 mM HEPES, 6 M urea, 1 μg/mL RapiGest SF Surfactant (Waters Corporation, Milford, MA, USA), and 1% (volume/volume) protease inhibitor (Thermo Scientific, Waltham, MA, USA). Total protein (20 μg) was transferred onto centrifugal filter units (Microcon-30 kDa; Merck, Darmstadt, Germany) for protein purification and tryptic digestion according to a Filter Aided Sample Preparation (FASP) protocol,^54^ with minor modifications. Briefly, the samples were washed with a buffer composed of 8 M urea and 100 mM Tris (pH 8.5). After the reduction with 8 mM dithiothreitol, the alkylation with 50 mM iodoacetamide and the removal of excess iodoacetamide with 8 mM dithiothreitol this washing step was repeated each time. Before adding trypsin (enzyme-to-protein ratio 1:50 (weight/weight)), the filter was washed with NH_4_HCO_3_ three times. After 16 h of incubation in a wet chamber at 37 °C, the peptides were washed from the filter with 50 mM NH_4_HCO_3_. Trifluoroacetic acid was added to create a final concentration of 1% (volume/volume). The samples were dried down at 45 °C and afterwards reconstituted in 3% acetonitrile and 0.1% formic acid in water to a final protein concentration of 150 ng/μL.

### Liquid Chromatography–Mass Spectrometry (LC–MS)

The tryptic peptides were analyzed on a nanoAcquity UPLC system coupled to a Synapt G2-Si HDMS mass spectrometer equipped with an electrospray ionization source (Waters Corporation, Manchester, UK). Mobile phase A contained 0.1% formic acid and 3% dimethyl sulfoxide in water and mobile phase B 0.1% formic acid and 3% dimethyl sulfoxide in acetonitrile. Protein (300 ng) was injected in trapping mode on a C18, 5 μm, 180 μm × 20 mm trap column (Waters Corporation). The following HSS-T3 C18 1.8 μm, 75 μm × 250 mm analytical column (Waters Corporation) was kept at 40 °C. For peptide separation, a gradient was run from 3–40% (volume/volume) mobile phase B over 120 min at a flow rate of 0.3 μL/min. The peptides were analyzed in positive ionization mode by using the UDMS^E^ approach published previously.^54,55^ A lock mass solution of [Glu1]-fibrinopeptide B (0.1 μM) and leu-enkephalin (1 μM) was introduced to the mass spectrometer via the reference sprayer every 60 s. The reproducibility and stability of the method was controlled with ten HeLa digest control samples (Thermo Scientific, Waltham, MA, USA) that were analyzed interspersed between the brain samples. A schematic illustration of the work flow is presented in Figure 2.

### LC–MS Data Processing and Label-Free Quantification Analysis

Raw data was processed using ProteinLynx Global Server (version 3.0.3, Waters Corporation, Milford, MA, USA). The database search against a randomized UniProt mouse database (UniProtKB version 14/01/2020) was done with a false discovery rate (FDR) of 0.01. Trypsin was set as the digest reagent. Carbamidomethyl cysteine was set as the fixed modification. Acetyl lysine, C-terminal amidation, asparagine deamidation, glutamine deamidation, and methionine oxidation were set as variable modifications. One missed cleavage per peptide was allowed. Minimum fragment ion matches per peptide were 1 and per protein were 3. Minimum peptide matches per protein were 2.

ISOQuant 1.8 was used for label-free quantification analysis.^54,55^ The workflow included nonlinear retention time alignment, signal clustering based on accurate mass, retention and drift time, annotation of signal clusters using PLGS identifications, intensity normalization, and protein isoform and homology filtering. The average intensity of the three most intense peptides of each protein was used for relative protein quantification (TOP3 quantification). The software settings are described in Table S6.

Results from ISOQuant were further analyzed for changes in protein levels in R 3.6.3 with the empirical Bayes method using the package limma.^56^ Histogram plots of the raw *p*-values can be found in Figure S2. Obtained *p*-values were adjusted for multiple comparisons with the package *q*-value, setting the FDR < 0.05.^57^ All log_2_-fold changes with an adjusted *p*-value < 0.05 were considered statistically significant. Please note that all figures and text in the results and discussion refer to adjusted *p*-values.

Altered protein levels were processed for GO enrichment analysis with Panther 15.0 using all identified proteins as reference.

### Western Blot Analysis

APPswe lysates from both SST-scFv8D3 treated and PBS treated groups, as well as WT controls, were mixed with LDS sample buffer (Thermo Scientific, Waltham, MA, USA) and Bolt sample reducing agent (Thermo Scientific, Waltham, MA, USA) and loaded onto Bolt 4–12% Bis-tris plus gels (Thermo Scientific, Waltham, MA, USA). Gels were run at 80 V for 1–2 h followed by transfer onto PVDF-membranes (Merck, Darmstadt, Germany). Membranes were blocked with 5% dry milk in TBS-Tween for 1 h at RT, followed by overnight staining at 4 °C with primary antibodies anti-syntaxin antibody (ab41453, Abcam, Cambridge, United Kingdom) and anti-β-actin antibody, clone AC-15 (Sigma-Aldrich, Stockholm, Sweden). The following day, membranes were washed with TBS-Tween buffer and incubated with goat antimouse IgG Alexa 680 (A21057, Life Technologies, Waltham, MA, USA) and donkey antirabbit IgG Alexa 800 (A32808, Invitrogen, Waltham, MA, USA) secondary antibodies for 1 h at RT. After they were washed three times with TBS-Tween buffer, signals were developed using the LI-COR odyssey machine (LI-COR biosciences, Homburg, Germany). Signal intensity was measured using Image Studio software (version 5.2.5). The measured signals from syntaxin were normalized against the loading control (β-actin).

### KC/GRO Quantification

Concentration of KC/GRO in the brain and plasma of SST-scFv8D3 treated and PBS treated APPswe mice, as well as WT controls, was quantified using a pro-inflammatory cytokine multiplex assay (K152QTD, Meso Scale Diagnostics, Rockville, MD, USA), according to the manufacturer protocol. Lysates from hippocampal area and rest of cerebrum were diluted at the 1:1 volume/volume ratio, while plasma samples were diluted at the 1:10 volume/volume ratio.

### Neuropeptide-Y and Substance-P Quantification

Brain concentrations of neuropeptide-Y and substance-P were quantified in treated APPswe mice as well as WT controls using ELISA, according to the manufacturer protocol (A3330/A80235, Antibodies.com, Cambridge, United Kingdom). Lysates from the hippocampal area and the rest of the cerebrum were diluted at the 1:5 volume/volume ratio.

### Statistical Analyses

Non-LC–MS results are presented as mean ± SD. The Shapiro-Wilk test was applied to test for normality. Data that passed the normality test were analyzed with one-way ANOVA followed by Bonferroni’s posthoc test. Data that did not pass the normality test were analyzed with the Kruskal–Wallis test followed by Dunn’s post hoc analysis. A significant *p*-value is defined as *p* < 0.05.

## Supporting Information Available

List of 1869 proteins quantified across all 42 samples, list of proteins with lower levels in hippocampus, list of proteins with higher levels in hippocampus, list of 2487 proteins quantified across all samples in hippocampus and rest of cerebrum, list of 94 hippocampal proteins that overlapped between 160 differentially expressed proteins in treatment study and 1283 differentially expressed proteins in transgenic study, ISOQuant 1.8 software settings used for label-free quantification analysis of LC–MS data, heatmap of differentially expressed hippocampal proteins, and histograms of raw *p*-values (PDF)
Results from ISOQuant were further analyzed for changes in protein levels in R 3.6.3 with empirical Bayes method using package limma (XLSX)
Average expression, *p*-value, adjusted *p*-value, *t*, *B*, significance, and log FC values for hippocampus and rest of cerebrum (XLSX)

## Notes

The mass spectrometry proteomics data have been deposited to the ProteomeXchange Consortium via the PRIDE partner repository with the data set identifiers PXD022221 and PXD026566. “dx.doi.org/10.6019/PXD022221 and dx.doi.org/10.6019/PXD026566”.

## Abbreviations

Aβ: amyloid-β
APLP-1: amyloid-like protein 1
APP: amyloid precursor protein
BBB: blood–brain barrier
FDR: false discovery rate
LC–MS: liquid chromatography–mass spectrometry
scFv: single chain fragment variable
SNARE: soluble *N*-ethylmaleimide-sensitive fusion protein attachment protein receptor
SST: somatostatin
TfR: transferrin receptor
WT: wild-type
