# Supplementary material for: Wide-Ranging Effects on the Brain Proteome in a Transgenic Mouse Model of Alzheimer’s Disease Following Treatment with a Brain-Targeting Somatostatin Peptide
Source: ACS Chem Neurosci. 2021 Jun 25;12(13):2529–41. doi: 10.1021/acschemneuro.1c00303 (PMC8291608; doi:10.1021/acschemneuro.1c00303)
Supplement: Supplementary file 1 — cn1c00303_si_001.pdf [file cn1c00303_si_001.pdf]

Supplementary Information

Wide-ranging effects on the brain proteome in a transgenic mouse model of Alzheimer's disease following treatment with a brain-targeting somatostatin peptide

Fadi Rofo[1], Friederike A. Sandbaumhüter[2], Aikaterini Chourlia[1], Nicole G. Metzendorf[1], Jamie I. Morrison[1], Stina Syvänen[3], Per E. Andrén[2,4], Erik T. Jansson[2], Greta Hultqvist[1]

1 Protein Drug Design, Faculty of Pharmacy, Biomedical Centre 591, Uppsala University, 75124, Uppsala, Sweden

2 Medical Mass Spectrometry, Department of Pharmaceutical Biosciences, Biomedical Centre 591, Uppsala University, 75124, Uppsala, Sweden.

3 Department of Public Health and Caring Sciences, Rudbeck Laboratory, Uppsala University, 75185, Uppsala, Sweden.

4 Science for Life Laboratory, Spatial Mass Spectrometry, Biomedical Centre 591, Uppsala University, 75124, Uppsala, Sweden.

Corresponding author: Greta Hultqvist; Email: greta.hultqvist@farmbio.uu.se; Phone: +46 70 2253522

Supplementary Table 1 List of 1869 proteins quantified across all 42 samples from SST-scFv8D3 treated and PBS treated APPswe mice using LC–MS (supplied as excel file).

Supplementary Table 2 List of proteins with lower levels in the hippocampus of SST-scFv8D3 treated APPswe compared to PBS treated APPswe mice

**UniProt ID**	**Protein description**	**Log2 fold change**	**Adjusted p-value**
WASF2_MOUSE	Wiskott-Aldrich syndrome protein family member 2	-1.91	0.0169
K1C17_MOUSE	Keratin, type I cytoskeletal 17	-1.75	0.0202
ACADV_MOUSE	Very long-chain specific acyl-CoA dehydrogenase, mitochondrial	-1.32	0.0266
PCP2_MOUSE	Purkinje cell protein 2	-1.10	0.0287
THIKA_MOUSE	3-ketoacyl-CoA thiolase A, peroxisomal	-1.06	0.0306
CLCB_MOUSE	Clathrin light chain B	-1.00	0.0346
SDHA_MOUSE	Succinate dehydrogenase [ubiquinone] flavoprotein subunit, mitochondrial	-0.99	0.0215
ODO2_MOUSE	Dihydrolipoyllysine-residue succinyltransferase component of 2-oxoglutarate dehydrogenase complex, mitochondrial	-0.98	0.0003

NPS3B_MOUSE	Protein NipSnap homolog 3B	-0.77	0.0427
RHG35_MOUSE	Rho GTPase-activating protein 35	-0.77	0.0107
NFS1_MOUSE	Cysteine desulfurase, mitochondrial	-0.76	0.0016
THIM_MOUSE	3-ketoacyl-CoA thiolase, mitochondrial	-0.75	0.0041
SCOT1_MOUSE	Succinyl-CoA:3-ketoacid coenzyme A transferase 1, mitochondrial	-0.75	0.0066
SSBP_MOUSE	Single-stranded DNA-binding protein, mitochondrial	-0.74	0.0238
PRDX3_MOUSE	Thioredoxin-dependent peroxide reductase, mitochondrial	-0.73	0.0001
APLP1_MOUSE	Amyloid-like protein 1	-0.72	0.0064
CHM4B_MOUSE	Charged multivesicular body protein 4b	-0.69	0.0452
GLSK_MOUSE	Glutaminase kidney isoform, mitochondrial	-0.69	0.0079
ACADM_MOUSE	Medium-chain specific acyl-CoA dehydrogenase, mitochondrial	-0.69	0.0052
CALR_MOUSE	Calreticulin	-0.68	0.0495
LMBL2_MOUSE	Lethal(3)malignant brain tumor-like protein 2	-0.68	0.0141
PYC_MOUSE	Pyruvate carboxylase, mitochondrial	-0.68	0.0485
HNRPF_MOUSE	Heterogeneous nuclear ribonucleoprotein F	-0.67	0.0365
IVD_MOUSE	Isovaleryl-CoA dehydrogenase, mitochondrial	-0.66	0.0016
K1C14_MOUSE	Keratin, type I cytoskeletal 14	-0.66	0.0141
UT14A_MOUSE	U3 small nucleolar RNA-associated protein 14 homolog A	-0.66	0.0263
GPX1_MOUSE	Glutathione peroxidase 1	-0.66	0.0333
EFTS_MOUSE	Elongation factor Ts, mitochondrial	-0.66	0.0141
THEM4_MOUSE	Acyl-coenzyme A thioesterase THEM4	-0.65	0.0257
ARSB_MOUSE	Arylsulfatase B	-0.65	0.0492
NFU1_MOUSE	NFU1 iron-sulfur cluster scaffold homolog, mitochondrial	-0.64	0.0477
ECHM_MOUSE	Enoyl-CoA hydratase, mitochondrial	-0.64	0.0083
ETFA_MOUSE	Electron transfer flavoprotein subunit alpha, mitochondrial	-0.64	0.0093
FAHD2_MOUSE	Fumarylacetoacetate hydrolase domain-containing protein 2A	-0.64	0.0028

IDHG1_MOUSE	Isocitrate dehydrogenase [NAD] subunit gamma 1, mitochondrial	-0.64	0.0066
ACADL_MOUSE	Long-chain specific acyl-CoA	-0.64	0.0083
CSPG5_MOUSE	Chondroitin sulfate proteoglycan 5	-0.63	0.0485
MANF_MOUSE	Mesencephalic astrocyte-derived neurotrophic factor	-0.62	0.0141
ERP29_MOUSE	Endoplasmic reticulum resident protein 29	-0.61	0.0108
ODO1_MOUSE	2-oxoglutarate dehydrogenase, mitochondrial	-0.60	0.0017
HCD2_MOUSE	3-hydroxyacyl-CoA dehydrogenase type-2	-0.60	0.0083
SUCB1_MOUSE	Succinate--CoA ligase [ADP-forming] subunit beta, mitochondrial	-0.60	0.0016
SSDH_MOUSE	Succinate-semialdehyde dehydrogenase, mitochondrial	-0.59	0.0016
ODPB_MOUSE	Pyruvate dehydrogenase E1 component subunit beta, mitochondrial	-0.59	0.0091
C1TM_MOUSE	Monofunctional C1-tetrahydrofolate synthase, mitochondria	-0.59	0.0041
ECH1_MOUSE	Delta(3,5)-Delta(2,4)-dienoyl-CoA isomerase, mitochondrial	-0.59	0.0106
CH10_MOUSE	10 kDa heat shock protein, mitochondrial	-0.58	0.0016
3HIDH_MOUSE	3-hydroxyisobutyrate dehydrogenase, mitochondrial	-0.58	0.0473
ETFB_MOUSE	Electron transfer flavoprotein subunit beta	-0.58	0.0016
THIL_MOUSE	Acetyl-CoA acetyltransferase, mitochondrial	-0.58	0.0025
DHB4_MOUSE	Peroxisomal multifunctional enzyme type 2	-0.57	0.0309
DGUOK_MOUSE	Deoxyguanosine kinase, mitochondrial	-0.57	0.0333
GRP75_MOUSE	Stress-70 protein, mitochondrial	-0.56	0.0016
GLU2B_MOUSE	Glucosidase 2 subunit beta	-0.56	0.0016
SUCA_MOUSE	Succinate--CoA ligase [ADP/GDP-forming] subunit alpha, mitochondrial	-0.56	0.0083
TSR2_MOUSE	Pre-rRNA-processing protein TSR2 homolog	-0.56	0.0169
NQO1_MOUSE	NAD(P)H dehydrogenase [quinone] 1	-0.55	0.0115
ABHDA_MOUSE	Mycophenolic acid acyl-glucuronide esterase, mitochondrial	-0.55	0.0323

ODP2_MOUSE	Dihydrolipoyllysine-residue acetyltransferase component of pyruvate dehydrogenase complex, mitochondrial	-0.54	0.0141
SNX6_MOUSE	Sorting nexin-6	-0.54	0.0141
FBX22_MOUSE	F-box only protein 22	-0.53	0.0477
CLPP_MOUSE	ATP-dependent Clp protease proteolytic subunit, mitochondrial	-0.53	0.0046
EFTU_MOUSE	Elongation factor Tu, mitochondrial	-0.52	0.0019
FUMH_MOUSE	Fumarate hydratase, mitochondrial	-0.52	0.0016
ALDH2_MOUSE	Aldehyde dehydrogenase, mitochondrial	-0.51	0.0066
MCCB_MOUSE	Methylcrotonoyl-CoA carboxylase beta chain, mitochondrial	-0.50	0.0452
CALU_MOUSE	Calumenin	-0.50	0.0087
SETD7_MOUSE	Histone-lysine N-methyltransferase SETD7	-0.49	0.0294
GAL3A_MOUSE	Glutamine amidotransferase-like class 1 domain-containing protein 3A, mitochondrial	-0.49	0.0083
EPMIP_MOUSE	EPM2A-interacting protein 1	-0.48	0.0064
CATA_MOUSE	Catalase	-0.48	0.0083
MMSA_MOUSE	Methylmalonate-semialdehyde dehydrogenase [acylating], mitochondrial	-0.48	0.0093
IDH3A_MOUSE	Isocitrate dehydrogenase [NAD] subunit alpha, mitochondrial	-0.48	0.0041
ODPX_MOUSE	Pyruvate dehydrogenase protein X component, mitochondrial	-0.48	0.0257
PSMA8_MOUSE	Proteasome subunit alpha type-8	-0.48	0.0202
SODM_MOUSE	Superoxide dismutase [Mn], mitochondrial	-0.48	0.0041
DLDH_MOUSE	Dihydrolipoyl dehydrogenase, mitochondrial	-0.47	0.0115
BLVRB_MOUSE	Flavin reductase (NADPH)	-0.46	0.0266
FABPH_MOUSE	Fatty acid-binding protein	-0.46	0.0351
RANG_MOUSE	Ran-specific GTPase-activating protein	-0.46	0.0126
PDIA1_MOUSE	Protein disulfide-isomerase	-0.45	0.0064
IDHP_MOUSE	Isocitrate dehydrogenase [NADP], mitochondrial	-0.45	0.0287
CATD_MOUSE	Cathepsin D	-0.44	0.0177
NIPS2_MOUSE	Protein NipSnap homolog 2	-0.43	0.0202

TPM3_MOUSE	Tropomyosin alpha-3 chain	-0.43	0.0438
IPYR2_MOUSE	Inorganic pyrophosphatase 2, mitochondrial	-0.43	0.0083
NEUL_MOUSE	Neurolysin, mitochondrial	-0.42	0.0141
PFD2_MOUSE	Prefoldin subunit 2	-0.41	0.0452
CH60_MOUSE	60 kDa heat shock protein, mitochondrial	-0.41	0.0117
LGUL_MOUSE	Lactoylglutathione lyase	-0.41	0.0169
AN32A_MOUSE	Acidic leucine-rich nuclear phosphoprotein 32 family member A	-0.41	0.0477
MDHM_MOUSE	Malate dehydrogenase, mitochondrial	-0.40	0.0083
KCRU_MOUSE	Creatine U-type, mitochondrial	-0.39	0.0290
AATM_MOUSE	Aspartate aminotransferase, mitochondrial	-0.39	0.0064
UBA3_MOUSE	NEDD8-activating enzyme E1 catalytic subunit	-0.39	0.0294
HIBCH_MOUSE	3-hydroxyisobutyryl-CoA hydrolase, mitochondrial	-0.38	0.0425
PPAC_MOUSE	Low molecular weight phosphotyrosine protein phosphatase	-0.38	0.0293
PDIA3_MOUSE	Protein disulfide-isomerase A3	-0.37	0.0064
RD23B_MOUSE	UV excision repair protein RAD23 homolog B	-0.36	0.0345
NACAM_MOUSE	Nascent polypeptide-associated complex subunit alpha	-0.35	0.0333
CISY_MOUSE	Citrate synthase, mitochondrial	-0.32	0.0141
ASGL1_MOUSE	Isoaspartyl peptidase/L-asparaginase	-0.32	0.0477
ACON_MOUSE	Aconitate hydratase, mitochondrial	-0.31	0.0096
PSB6_MOUSE	Proteasome subunit beta type-6	-0.29	0.0287
CNDP2_MOUSE	Cytosolic non-specific dipeptidase	-0.27	0.0338

Supplementary Table 3 List of proteins with higher levels in the hippocampus of SST-scFv8D3 treated APPswe compared to PBS treated APPswe mice

UniProt ID	Protein description	Log2 fold change	Adjusted p-value
CDK20_MOUSE	Cyclin-dependent kinase 20	1.45	0.0018
ATLA1_MOUSE	Atlastin-1	1.33	0.0091
NCDN_MOUSE	Neurochondrin	1.30	0.0038
GSTM3_MOUSE	Glutathione S-transferase Mu 3	1.21	0.0190
MP2K2_MOUSE	Dual specificity mitogen-activated protein kinase 2	1.10	0.0257
MP2K1_MOUSE	Dual specificity mitogen-activated protein kinase 1	1.08	0.0185
YKT6_MOUSE	Synaptobrevin homolog YKT6	1.06	0.0169
VIAAT_MOUSE	Vesicular inhibitory amino acid transporter	1.02	0.0171
2ABG_MOUSE	Serine/threonine-protein phosphatase 2A 55 kDa regulatory subunit B gamma isoform	0.99	0.0126
CAND1_MOUSE	Cullin-associated NEDD8-dissociated protein 1	0.97	0.0338
2A5E_MOUSE	Serine/threonine-protein phosphatase 2A 56 kDa regulatory subunit epsilon isoform	0.95	0.0078
STX1A_MOUSE	Syntaxin-1A	0.92	0.0072
DYN1_MOUSE	Dynamin-1	0.89	0.0107
SC22B_MOUSE	Vesicle-trafficking protein SEC22b	0.89	0.0064
DCTN1_MOUSE	Dynactin subunit 1	0.85	0.0155
FA49A_MOUSE	CYFIP-related Rac1 interactor A	0.85	0.0083
PDC6I_MOUSE	Programmed cell death 6-interacting protein	0.82	0.0153
FARP1_MOUSE	FERM, ARHGEF and pleckstrin domain-containing protein 1	0.81	0.0091
PRUN1_MOUSE	Exopolyphosphatase PRUNE1	0.81	0.0202
EIF3L_MOUSE	Eukaryotic translation initiation factor 3 subunit L	0.81	0.0186
CADM3_MOUSE	Cell adhesion molecule 3	0.80	0.0083
RNF14_MOUSE	E3 ubiquitin-protein ligase RNF14	0.78	0.0360
VAPA_MOUSE	Vesicle-associated membrane protein-associated protein A	0.77	0.0401
DIRA1_MOUSE	GTP-binding protein Di-Ras1	0.77	0.0184
DYN3_MOUSE	Dynamin-3	0.75	0.0323
CAPS1_MOUSE	Calcium-dependent secretion activator 1	0.75	0.0455
CAP1_MOUSE	Adenylyl cyclase-associated protein 1	0.72	0.0141
ATX10_MOUSE	Ataxin-10	0.72	0.0237

AP3S1_MOUSE	AP-3 complex subunit sigma-1	0.71	0.0179
PCBP2_MOUSE	Poly(rC)-binding protein 2	0.70	0.0083
FA98B_MOUSE	Protein FAM98B	0.69	0.0416
AP3M2_MOUSE	AP-3 complex subunit mu-2	0.69	0.0141
PCBP1_MOUSE	Poly(rC)-binding protein 1	0.68	0.0242
TPM2_MOUSE	Tropomyosin beta chain	0.66	0.0477
CUL2_MOUSE	Cullin-2	0.66	0.0416
DP13A_MOUSE	DCC-interacting protein 13-alpha	0.65	0.0093
RTN3_MOUSE	Reticulon-3	0.65	0.0309
COPD_MOUSE	Coatomer subunit delta	0.64	0.0371
RTN1_MOUSE	Reticulon-1	0.64	0.0266
MP2K4_MOUSE	Dual specificity mitogen-activated protein kinase 4	0.63	0.0427
CN37_MOUSE	2',3'-cyclic-nucleotide 3'-phosphodiesterase	0.62	0.0351
IMB1_MOUSE	Importin subunit beta-1	0.62	0.0083
HPCL4_MOUSE	Hippocalcin-like protein 4	0.62	0.0293
VPP1_MOUSE	V-type proton ATPase 116 kDa subunit a isoform 1	0.60	0.0477
MK04_MOUSE	Mitogen-activated protein kinase 4	0.60	0.0177
BRSK2_MOUSE	Serine/threonine-protein kinase BRSK2	0.53	0.0091
SYT1_MOUSE	Synaptotagmin-1	0.50	0.0295
NECP1_MOUSE	Adaptin ear-binding coat-associated protein 1	0.50	0.0486
RTN4_MOUSE	Reticulon-4	0.49	0.0294
IMA4_MOUSE	Importin subunit alpha-4	0.45	0.0338
DC1L1_MOUSE	Cytoplasmic dynein 1 light intermediate chain 1	0.45	0.0482
PDE2A_MOUSE	cGMP-dependent 3',5'-cyclic phosphodiesterase	0.44	0.0177
RHG01_MOUSE	Rho GTPase-activating protein 1	0.41	0.0202
CSN6_MOUSE	COP9 signalosome complex subunit 6	0.37	0.0427
CAP2_MOUSE	Adenylyl cyclase-associated protein 2	0.31	0.0400

Supplementary Table 4 List of 2487 proteins quantified across all samples in the hippocampus and rest of cerebrum from untreated APPswe and untreated WT mice using LC–MS (supplied as excel file).

Supplementary Table 5 List of 94 hippocampal proteins that overlapped between the 160 differentially expressed proteins in the treatment study and the 1283 differentially expressed proteins in the transgenic study. Proteins that are shifted towards WT levels following SST-scFv8D3 treatment are marked with ✓

**UniProt ID**	**APPswe (SST-scFv8D3) vs APPswe (PBS)**	**APPswe vs WT**	**Treatment with SST-scFv8D3**

	Log2 fold change	Adjusted p-value	Log2 fold change	Adjusted p-value	shifts the altered protein towards WT levels (✓)
MDHM_MOUSE	-0.40	0.0083	0.79	0.0095	✓
THIL_MOUSE	-0.58	0.0025	1.34	0.0076	✓
CH10_MOUSE	-0.58	0.0016	1.25	0.0149	✓
CISY_MOUSE	-0.32	0.0141	0.66	0.0329	✓
FUMH_MOUSE	-0.52	0.0016	1.12	0.0064	✓
PDIA3_MOUSE	-0.37	0.0064	-1.50	<0.0001	
SUCB1_MOUSE	-0.60	0.0016	1.15	0.0194	✓
KCRU_MOUSE	-0.39	0.0290	-0.61	0.0278	
SODM_MOUSE	-0.48	0.0041	1.12	0.0152	✓
ETFA_MOUSE	-0.64	0.0093	1.71	0.0017	✓
LGUL_MOUSE	-0.41	0.0169	-0.57	0.0042	
CN37_MOUSE	0.62	0.0351	3.07	<0.0001	
STX1A_MOUSE	0.92	0.0072	7.26	<0.0001	
CALR_MOUSE	-0.68	0.0495	-0.76	0.0006	
SYT1_MOUSE	0.50	0.0295	4.03	<0.0001	
FABPH_MOUSE	-0.46	0.0351	-0.54	0.0205	
IDHG1_MOUSE	-0.64	0.0066	1.02	0.0250	✓
HPCL4_MOUSE	0.62	0.0293	1.10	0.0132	
NACAM_MOUSE	-0.35	0.0333	-2.18	<0.0001	
GAL3A_MOUSE	-0.49	0.0083	1.74	0.0036	✓
PRDX3_MOUSE	-0.73	0.0001	1.08	0.0300	✓
NCDN_MOUSE	1.30	0.0038	-1.82	0.0076	✓
THIM_MOUSE	-0.75	0.0041	1.13	0.0048	✓
CLCB_MOUSE	-1.00	0.0346	1.67	0.0013	✓
AN32A_MOUSE	-0.41	0.0477	-2.23	<0.0001	
PDIA1_MOUSE	-0.45	0.0064	-0.99	0.0001	
ECHM_MOUSE	-0.64	0.0083	1.78	0.0016	✓
IPYR2_MOUSE	-0.43	0.0083	1.69	0.0016	✓
RD23B_MOUSE	-0.36	0.0345	-0.62	0.0119	
SSDH_MOUSE	-0.59	0.0016	1.13	0.0239	✓

MP2K4_MOUSE	0.63	0.0427	-0.72	0.0371	✓
IDHP_MOUSE	-0.45	0.0287	1.64	0.0006	✓
SUCA_MOUSE	-0.56	0.0083	1.29	0.0168	✓
MMSA_MOUSE	-0.48	0.0093	1.10	0.0032	✓
ETFB_MOUSE	-0.58	0.0016	1.86	0.0011	✓
ACADL_MOUSE	-0.64	0.0083	0.85	0.0293	✓
PSMA8_MOUSE	-0.48	0.0202	-2.47	0.0015	
NQO1_MOUSE	-0.55	0.0115	-1.46	0.0273	
HCD2_MOUSE	-0.60	0.0083	1.77	0.0076	✓
CAP2_MOUSE	0.31	0.0400	-0.48	0.0468	✓
FAHD2_MOUSE	-0.64	0.0028	2.01	0.0075	✓
MP2K1_MOUSE	1.08	0.0185	-1.62	0.0138	✓
3HIDH_MOUSE	-0.58	0.0473	1.31	0.0034	✓
PCBP1_MOUSE	0.68	0.0242	-1.70	0.0005	✓
IVD_MOUSE	-0.66	0.0016	1.27	0.0044	✓
CAPS1_MOUSE	0.75	0.0455	1.33	0.0056	
VPP1_MOUSE	0.60	0.0477	3.65	<0.0001	
PCBP2_MOUSE	0.70	0.0083	-2.07	<0.0001	✓
FA49A_MOUSE	0.85	0.0083	-2.05	0.0117	✓
GPX1_MOUSE	-0.66	0.0333	0.57	0.0160	✓
RHG01_MOUSE	0.41	0.0202	2.33	<0.0001	
NECP1_MOUSE	0.50	0.0486	1.79	<0.0001	
CATD_MOUSE	-0.44	0.0177	1.30	0.0095	✓
THEM4_MOUSE	-0.65	0.0257	1.31	0.0139	✓
TSR2_MOUSE	-0.56	0.0169	-0.50	0.0201	
EFTS_MOUSE	-0.66	0.0141	2.35	0.0043	✓
ERP29_MOUSE	-0.61	0.0108	-0.54	0.0268	
CALU_MOUSE	-0.50	0.0087	-0.77	0.0024	
RTN4_MOUSE	0.49	0.0294	0.89	0.0229	
BLVRB_MOUSE	-0.46	0.0266	0.59	0.0486	✓
RTN1_MOUSE	0.64	0.0266	-1.53	0.0014	✓
DC1L1_MOUSE	0.45	0.0482	-1.21	0.0169	✓
NIPS2_MOUSE	-0.43	0.0202	0.96	0.0095	✓

HIBCH_MOUSE	-0.38	0.0425	1.90	0.0029	✓
CHM4B_MOUSE	-0.69	0.0452	-2.95	<0.0001	
GLU2B_MOUSE	-0.56	0.0016	-0.89	0.0026	
2A5E_MOUSE	0.95	0.0078	2.08	0.0006	
VIAAT_MOUSE	1.02	0.0171	3.26	<0.0001	
SDHA_MOUSE	-0.99	0.0215	3.06	0.0017	✓
NFS1_MOUSE	-0.76	0.0016	1.17	0.0419	✓
THIKA_MOUSE	-1.06	0.0306	1.23	0.0017	✓
NPS3B_MOUSE	-0.77	0.0427	2.31	0.0065	✓
CLPP_MOUSE	-0.53	0.0046	3.50	<0.0001	✓
ACADM_MOUSE	-0.69	0.0052	0.63	0.0448	✓
NFU1_MOUSE	-0.64	0.0477	0.98	0.0447	✓
ARSB_MOUSE	-0.65	0.0492	0.89	0.0371	✓
MCCB_MOUSE	-0.50	0.0452	1.18	0.0085	✓
NEUL_MOUSE	-0.42	0.0141	0.63	0.0294	✓
LMBL2_MOUSE	-0.68	0.0141	2.06	0.0258	✓
DGUOK_MOUSE	-0.57	0.0333	1.89	0.0036	✓
FARP1_MOUSE	0.81	0.0091	-1.11	0.0127	✓
PDE2A_MOUSE	0.44	0.0177	0.77	0.0211	
FA98B_MOUSE	0.69	0.0416	-1.99	0.0184	✓
COPD_MOUSE	0.64	0.0371	-0.76	0.0093	✓
FBX22_MOUSE	-0.53	0.0477	-1.22	0.0003	
MANF_MOUSE	-0.62	0.0141	0.77	0.0079	✓
CATA_MOUSE	-0.48	0.0083	1.41	0.0007	✓
RNF14_MOUSE	0.78	0.0360	-1.59	0.0112	✓
DP13A_MOUSE	0.65	0.0093	-1.19	0.0434	✓
ATX10_MOUSE	0.72	0.0237	-1.94	0.0066	✓
ABHDA_MOUSE	-0.55	0.0323	2.68	0.0014	✓
AP3S1_MOUSE	0.71	0.0179	1.27	0.0028	
C1TM_MOUSE	-0.59	0.0041	2.06	0.0065	✓
SC22B_MOUSE	0.89	0.0064	-1.26	0.0181	✓

Supplementary Table 6 ISOQuant 1.8 software settings used for label-free quantification analysis of LC–MS data

parameter	value
isoquant.pluginQueue.name	design project and run ISOQuant analysis
process.peptide.deplete.PEP_FRAG_2	false
process.peptide.deplete.CURATED_0	false
process.peptide.statistics.doSequenceSearch	false
process.emrt.minIntensity	1000
process.emrt.minMass	500
process.emrt.rt.alignment.match.maxDeltaMass.ppm	10

process.emrt.rt.alignment.match.maxDeltaDriftTime	2
process.emrt.rt.alignment.normalizeReferenceTime	false
process.emrt.rt.alignment.maxProcesses	24
process.emrt.rt.alignment.referenceRun.selectionMethod	AUTO
process.emrt.clustering.preclustering.orderSequence	MTMTMT
process.emrt.clustering.preclustering.maxDistance.mass.ppm	6.06E-6
process.emrt.clustering.preclustering.maxDistance.time.min	0.202
process.emrt.clustering.preclustering.maxDistance.drift	2.02
process.emrt.clustering.distance.unit.mass.ppm	6.0E-6
process.emrt.clustering.distance.unit.time.min	0.2
process.emrt.clustering.distance.unit.drift.bin	2
process.emrt.clustering.dbscan.minNeighborCount	2
process.identification.peptide.minReplicationRate	2
process.identification.peptide.minScore	0
process.identification.peptide.minOverallMaxScore	0
process.identification.peptide.minSequenceLength	6
process.identification.peptide.acceptType.PEP_FRAG_1	true
process.identification.peptide.acceptType.IN_SOURCE	false
process.identification.peptide.acceptType.MISSING_CLEAVAGE	false
process.identification.peptide.acceptType.NEUTRAL_LOSS_H20	false
process.identification.peptide.acceptType.NEUTRAL_LOSS_NH3	false
process.identification.peptide.acceptType.PEP_FRAG_2	false
process.identification.peptide.acceptType.DDA	true
process.identification.peptide.acceptType.VAR_MOD	false
process.identification.peptide.acceptType.PTM	false
process.annotation.peptide.maxSequencesPerEMRTCluster	1
process.annotation.protein.resolveHomology	true
process.annotation.peptide.maxFDR	0.01
process.annotation.useSharedPeptides	all
process.normalization.lowess.bandwidth	0.3
process.normalization.orderSequence	XPIR

process.normalization.minIntensity	3000
process.quantification.peptide.minMaxScorePerCluster	0
process.quantification.peptide.acceptType.IN_SOURCE	false
process.quantification.peptide.acceptType.MISSING_CLEAVAGE	false
process.quantification.peptide.acceptType.NEUTRAL_LOSS_H20	false
process.quantification.peptide.acceptType.NEUTRAL_LOSS_NH3	false
process.quantification.peptide.acceptType.PEP_FRAG_1	true
process.quantification.peptide.acceptType.PEP_FRAG_2	false
process.quantification.peptide.acceptType.VAR_MOD	false
process.quantification.peptide.acceptType.PTM	false
process.quantification.peptide.acceptType.DDA	true
process.quantification.topx.degree	3
process.quantification.topx.allowDifferentPeptides	true
process.quantification.minPeptidesPerProtein	1
process.quantification.topx.allowDifferentPeptides	true
process.quantification.maxProteinFDR	0.01

Supplementary Figure 1

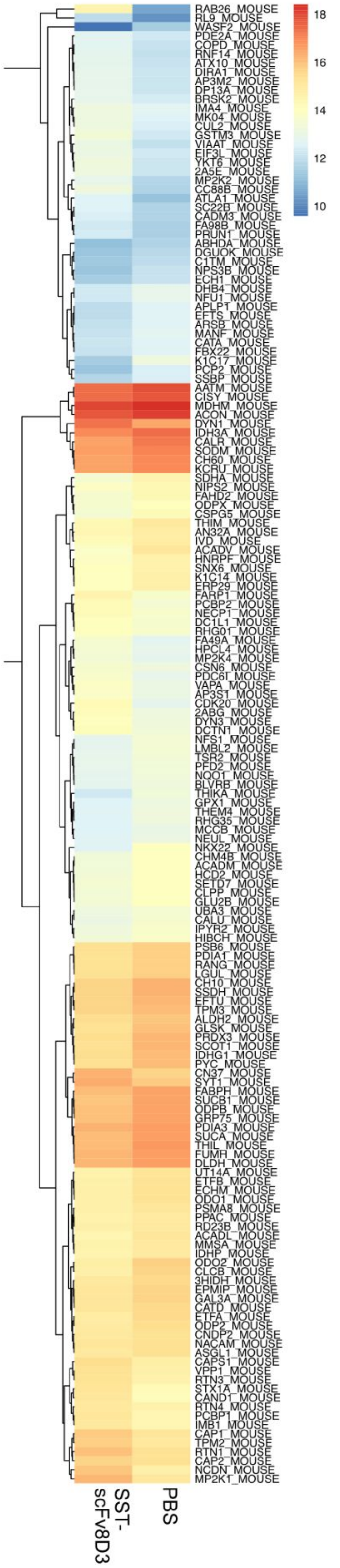
RAB26_MOUSE
RL9_MOUSE
WASF2_MOUSE
PDE2A_MOUSE
COPD_MOUSE
RNF14_MOUSE
ATX10_MOUSE
DIRA1_MOUSE
AP3M2_MOUSE
DP13A_MOUSE
BRSK2_MOUSE
IMA4_MOUSE
MK04_MOUSE
CUL2_MOUSE
GSTM3_MOUSE
VIAAT_MOUSE
EIF3L_MOUSE
YKT6_MOUSE
2A5E_MOUSE
MP2K2_MOUSE
CC88B_MOUSE
ATLA1_MOUSE
SC22B_MOUSE
CADM3_MOUSE
FA98B_MOUSE
PRUN1_MOUSE
ABHDA_MOUSE
DGUOK_MOUSE
C1TM_MOUSE
NPS3B_MOUSE
ECH1_MOUSE
DHB4_MOUSE
NFU1_MOUSE
APLP1_MOUSE
EFTS_MOUSE
ARSB_MOUSE
MANF_MOUSE
CATA_MOUSE
FBX22_MOUSE
K1C17_MOUSE
PCP2_MOUSE
SSBP_MOUSE
AATM_MOUSE
CISY_MOUSE
MDHM_MOUSE
ACON_MOUSE
DYN1_MOUSE
IDH3A_MOUSE
CALR_MOUSE
SODM_MOUSE
CH60_MOUSE
KCRU_MOUSE
SDHA_MOUSE
NIPS2_MOUSE
FAHD2_MOUSE
ODPX_MOUSE
CSPG5_MOUSE
THIM_MOUSE
AN32A_MOUSE
IVD_MOUSE
ACADV_MOUSE
HNRPF_MOUSE
SNX6_MOUSE
K1C14_MOUSE
ERP29_MOUSE
FARP1_MOUSE
PCBP2_MOUSE
NECP1_MOUSE
DC1L1_MOUSE
RHG01_MOUSE
FA49A_MOUSE
HPCL4_MOUSE
MP2K4_MOUSE
CSN6_MOUSE
PDC6I_MOUSE
VAPA_MOUSE
AP3S1_MOUSE
CDK20_MOUSE
2ABG_MOUSE
DYN3_MOUSE
DCTN1_MOUSE
NFS1_MOUSE
LMBL2_MOUSE
TSR2_MOUSE
PFD2_MOUSE
NQO1_MOUSE
BLVRB_MOUSE
THIKA_MOUSE
GPX1_MOUSE
THEM4_MOUSE
RHG35_MOUSE
MCCB_MOUSE
NEUL_MOUSE
NKX22_MOUSE
CHM4B_MOUSE
ACADM_MOUSE
HCD2_MOUSE
SETD7_MOUSE
CLPP_MOUSE
GLU2B_MOUSE
UBA3_MOUSE
CALU_MOUSE
IPYR2_MOUSE
HIBCH_MOUSE
PSB6_MOUSE
PDIA1_MOUSE
RANG_MOUSE
LGUL_MOUSE
CH10_MOUSE
SSDH_MOUSE
EFTU_MOUSE
TPM3_MOUSE
ALDH2_MOUSE
GLSK_MOUSE
PRDX3_MOUSE
SCOT1_MOUSE
IDHG1_MOUSE
PYC_MOUSE
CN37_MOUSE
SYT1_MOUSE
FABPH_MOUSE
SUCB1_MOUSE
ODPB_MOUSE
GRP75_MOUSE
PDIA3_MOUSE
SUCA_MOUSE
THIL_MOUSE
FUMH_MOUSE
DLDH_MOUSE
UT14A_MOUSE
ETFB_MOUSE
ECHM_MOUSE
ODO1_MOUSE
PSMA8_MOUSE
PPAC_MOUSE
RD23B_MOUSE
ACADL_MOUSE
MMSA_MOUSE
IDHP_MOUSE
ODO2_MOUSE
CLCB_MOUSE
3HIDH_MOUSE
EPMIP_MOUSE
GAL3A_MOUSE
CATD_MOUSE
ETFA_MOUSE
ODP2_MOUSE
CNDP2_MOUSE
NACAM_MOUSE
ASGL1_MOUSE
CAPS1_MOUSE
VPP1_MOUSE
RTN3_MOUSE
STX1A_MOUSE
CAND1_MOUSE
RTN4_MOUSE
PCBP1_MOUSE
IMB1_MOUSE
CAP1_MOUSE
TPM2_MOUSE
RTN1_MOUSE
CAP2_MOUSE
NCDN_MOUSE
MP2K1_MOUSE
18
16
14
12
10
SST-scFv8D3
PBS

Supplementary Figure 1 Heatmap of the differentially expressed hippocampal proteins between SST-scFv8D3 treated and PBS treated APPswe mice.

Supplementary Figure 2

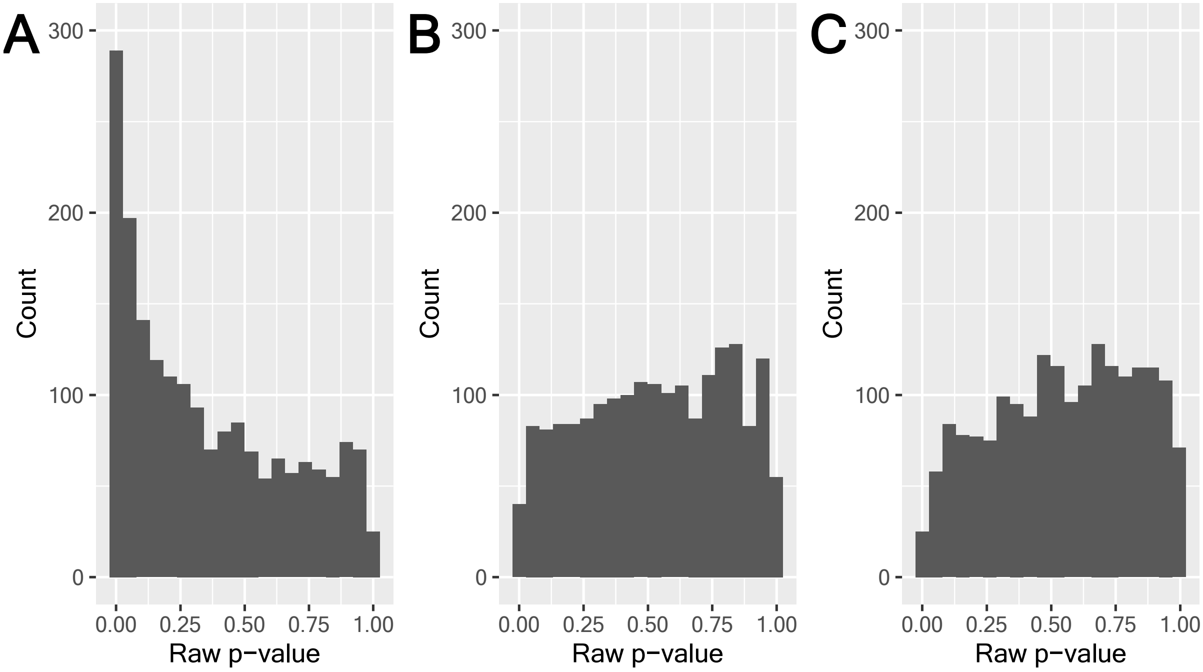

Supplementary Figure 2 Histograms of the raw p-values (before multiple testing correction) of the 1869 proteins quantified using LC–MS in the hippocampus (**A**), rest of cerebrum (**B**) and cerebellum (**C**) of SST-scFv8D3 treated APPswe mice compared to PBS treated group.
